# HSF5 Deficiency Causes Male Infertility Involving Spermatogenic Arrest at Meiotic Prophase I in Humans and Mice

**DOI:** 10.1002/advs.202402412

**Published:** 2024-07-03

**Authors:** Mohan Liu, Lingbo Wang, Yifei Li, Erlei Zhi, Gan Shen, Xiaohui Jiang, Dingming Li, Xinya Zhao, Tiechao Ruan, Chuan Jiang, Xiang Wang, Xueguang Zhang, Yanjiang Zheng, Bangguo Wu, Ningjing Ou, Guicheng Zhao, Siyu Dai, Ruixi Zhou, Li Yang, Yihong Yang, Hanmin Liu, Ying Shen

**Affiliations:** ^1^ Department of Obstetrics/Gynecology Gynecologic and Pediatric Diseases and Birth Defects of Ministry of Education West China Second University Hospital Sichuan University Chengdu 610041 China; ^2^ Department of Biotherapy Cancer Center and State Key Laboratory of Biotherapy West China Hospital Sichuan University Chengdu 610041 China; ^3^ Shanghai Key Laboratory of Metabolic Remodeling and Health Institute of Metabolism and Integrative Biology Institute of Reproduction and Development Obstetrics and Gynecology Hospital Fudan University Shanghai 200433 China; ^4^ Key Laboratory of Birth Defects and Related Diseases of Women and Children of MOE Department of Pediatrics West China Second University Hospital Sichuan University Chengdu 610041 China; ^5^ Urology Urologic Medical Center Shanghai General Hospital Shanghai Jiao Tong University School of Medicine Shanghai 200000 China; ^6^ Human Sperm Bank Key Laboratory of Birth Defects and Related Diseases of Women and Children Ministry of Education West China Second University Hospital Sichuan University Chengdu 610041 China; ^7^ NHC Key Laboratory of Chronobiology Sichuan University Chengdu 610041 China; ^8^ West China School of preclinical medicine and forensic medicine Sichuan University Chengdu 610041 China; ^9^ Department of Pediatrics West China Second University Hospital Sichuan University Chengdu 610041 China; ^10^ Department of Urology The First Affiliated Hospital of Chongqing Medical University Chongqing 400016 China; ^11^ Department of Pediatric Pulmonology and Immunology West China Second University Hospital Sichuan University Chengdu 610041 China; ^12^ Reproduction Medical Center of West China Second University Hospital Key Laboratory of Obstetric Gynecologic and Pediatric Diseases and Birth Defects of Ministry of Education Sichuan University Chengdu 610041 China

**Keywords:** gene therapy, HSF5, male infertility, meiotic arrest, transcription

## Abstract

Meiosis is a specialized cell division process that generates gametes for sexual reproduction. However, the factors and underlying mechanisms involving meiotic progression remain largely unknown, especially in humans. Here, it is first showed that HSF5 is associated with human spermatogenesis. Patients with a pathogenic variant of *HSF5* are completely infertile. Testicular histologic findings in the patients reveal rare postmeiotic germ cells resulting from meiotic prophase I arrest. *Hsf5* knockout (KO) mice confirms that the loss of HSF5 causes defects in meiotic recombination, crossover formation, sex chromosome synapsis, and sex chromosome inactivation (MSCI), which may contribute to spermatocyte arrest at the late pachytene stage. Importantly, spermatogenic arrest can be rescued by compensatory HSF5 adeno‐associated virus injection into KO mouse testes. Mechanistically, integrated analysis of RNA sequencing and chromatin immunoprecipitation sequencing data revealed that HSF5 predominantly binds to promoters of key genes involved in crossover formation (e.g., *HFM1*, *MSH5* and *MLH3*), synapsis (e.g., *SYCP1*, *SYCP2* and *SYCE3*), recombination (*TEX15*), and MSCI (*MDC1*) and further regulates their transcription during meiotic progression. Taken together, the study demonstrates that HSF5 modulates the transcriptome to ensure meiotic progression in humans and mice. These findings will aid in genetic diagnosis of and potential treatments for male infertility.

## Graphical abstract

The findings identify *HSF5* as a novel genetic cause for human male infertility associated with meiotic arrest. Moreover, the molecular mechanism of *HSF5* is first unambiguously elucidated as a transcriptional regulator orchestrating the transcriptome to ensure meiotic progression in humans and mice. This study sheds light on the genetic diagnosis and potential treatment for male infertility.

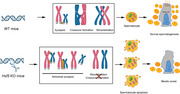

## 1 Introduction

Approximately 15% of reproductive‐aged couples worldwide suffer from infertility, which is a serious medical issue as well as a major social problem.^[^ ^1^ ^]^ Male infertility accounts for half of cases of infertility, and spermatogenic defects play a major role in these cases.^[^ ^2^ ^]^ Spermatogenesis is a carefully orchestrated developmental process in which diploid spermatogonial stem cells produce mature spermatozoa via three main phases: mitosis, meiosis and spermiogenesis. Specifically, meiosis is a conserved and special type of cell division that generates gametes for sexual reproduction.^[^ ^3^ ^]^ Although artificial assisted reproductive technology, especially intracytoplasmic sperm injection (ICSI) treatment, has enabled an increasing number of infertile males to father biologic children, men with meiotic defects do not benefit from these techniques due to the low likelihood of obtaining sperm through surgical testicular sperm extraction procedures.^[^ ^4^ ^]^ Thus, studies addressing the fundamental understanding of the mechanisms controlling meiosis can provide valuable information for the clinical diagnosis and treatment of male infertility involving meiotic arrest.

Meiosis prophase I, a key phase of meiosis, comprises an ordered series of events, including the programmed formation of DNA double‐strand breaks (DSBs), homologous chromosome pairing, synapsis, interhomologous recombination, and crossover formation.^[^ ^5^ ^]^ Meiosis prophase I (lasting ≈ 13 days in mice), which is divided into five substages (leptotene, zygotene, pachytene, diplotene, and diakinesis) takes longer than mitotic prophase to provide sufficient time for completion of these events.^[^ ^6^ ^]^ Pachytene, which is required for the acquisition of metaphase competence, is the longest stage and lasts six days in male mice.^[^ ^7^, ^8^ ^]^ At least 2300 genes are highly enriched in meiotic cells in humans and mice, and various mutant mouse models have been developed for studying key molecular aspects of prophase I over the past decades.^[^ ^9^, ^10^, ^11^ ^]^ However, the machineries modulating this process remain poorly understood, especially in humans.

Heat shock transcription factor 5 (HSF5) is a member of the HSF family, which are well known transcriptional regulators of genes encoding heat shock proteins as well as other types of proteins and have functions in reproduction, the immune response and the aging process.^[^ ^12^ ^]^ The expression of the HSF5 protein is restricted to spermatocytes and round spermatids in human and rat testes and is also enriched in mouse testes.^[^ ^13^ ^]^ Therefore, HSF5 is putatively associated with spermatogenesis due to its testis‐specific expression pattern. Consistent with this idea, a study in zebrafish showed that mutant *hsf5* ^−/−^ males were infertile, with a drastic reduction in sperm count and a marked increase in abnormal sperm morphology; these observations indicated the importance of Hsf5 in the early stages of spermatogenesis, specifically progression through meiotic prophase 1, in zebrafish.^[^ ^14^ ^]^ Although previous studies have indicated that HSF5 might be involved in male fecundity, the exact role of HSF5 in meiosis in mammals, and especially in humans and mice, is still unclear, as is the underlying mechanism by which HSFs mediate spermatogenesis.

In the present study, we identified a novel homozygous missense mutation, c.586 C > T [p.R196C] in *HSF5* in two infertile siblings from a consanguineous family by whole‐exome sequencing (WES). The two affected patients exhibited a cryptozoospermia phenotype, and testicular histological analysis detected almost no round or elongated spermatids. We further demonstrated that in mice, the loss of HSF5 is associated with abnormal meiotic recombination, synapsis, crossover, and meiotic sex chromosome inactivation (MSCI), leading to meiotic arrest at the late pachytene stage and ultimately male infertility. Furthermore, chromatin immunoprecipitation sequencing (ChIP‐seq) and RNA sequencing (RNA‐seq) indicated that HSF5 predominantly binds to the promoters of meiosis‐related genes and maintains their expression in the late pachytene stage. To our knowledge, this is the first report to reveal that *HSF5* functions as a transcriptional regulator of spermatogenesis in humans and mice and that the loss of HSF5 is a pathogenic factor for male infertility.

## 2 Results

### 2.1 Identification of a Pathogenic Variant in *HSF5* in Infertile Men

**Figure 1:**
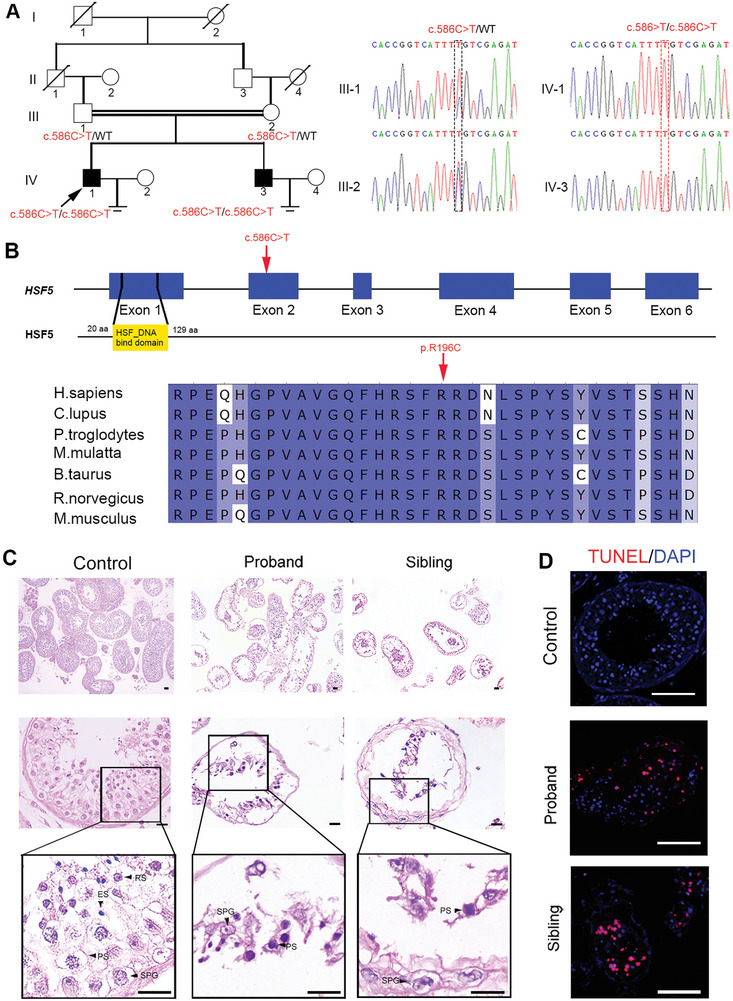
An *HSF5* mutation in a family with meiotic arrest and male infertility. A) Pedigree structure of the infertile family. Squares represent male pedigree members, circles represent female pedigree members, solid symbols represent affected infertile pedigree members, and hollow symbols represent unaffected pedigree members. The proband is marked with a black arrow. Sequence chromatograms for the identified *HSF5* mutation in this family are shown. The homozygotes are indicated by the red box, and the heterozygotes are indicated by the black box. B) Pattern diagram of the location of the variant in the human *HSF5* gene and HSF5 protein; the variation site is indicated by a red arrow. The site of the *HSF5* missense variant is evolutionarily conserved among different species. C) Histology of seminiferous tubules from controls and patients. H&E staining revealed sparse and disordered SPG and PS and nearly no postmeiotic cells in the patient testes compared to those in the control testes. The black arrows indicate the different types of spermatogenic cells. SPG, spermatogonium; PS, primary spermatocyte; RS, round spermatid; ES, elongating/elongated spermatid (Scale bars, 125 µm). D) Immunofluorescence staining showed an increase in TUNEL‐stained cells in the seminiferous tubules of the patients (Blue, DAPI; red, TUNEL; scale bars, 5 µm).

A 29‐year‐old man (IV‐1) from a consanguineous family who had been diagnosed with primary infertility for three years was recruited for our study (**Figure** **1**A). His somatic cell karyotype (46, XY) and hormone levels were normal, and no azoospermia factor (AZF) microdeletion was detected. Routine semen assessments were conducted following the WHO guidelines, and no spermatozoa were initially observed in the replicated wet preparations. His sibling (IV‐3) was also affected (Figure 1A), failing to conceive with regular unprotected sexual intercourse. To explore the genetic cause of the sterility in this family, we performed WES analysis on the two siblings. A novel homozygous missense mutation, c.586 C > T [p.R196C], in *HSF5* was identified in both patients (Figure 1A). Subsequent Sanger sequencing performed on other family members indicated that the patients’ unaffected parents (III‐1 and III‐2) harbored a heterozygous variant of c.586 C > T [p.R196C] in *HSF5*, suggesting an autosomal recessive inheritance pattern (Figure 1A). Notably, prevalence analysis in the 1000 Genomes, ExAC and gnomAD databases revealed that this variant was rare in the general population, as it was either detected at an extremely low allele frequency or absent (Table S1, Supporting Information), and neither heterozygous nor homozygous variants of *HSF5* (c.586 C > T) were detected in our cohort of 1000 Chinese control men. Moreover, the R196 of HSF5 was highly conserved among multiple species (Figure 1B) and the R196C mutation was predicted to be damaging via in silico bioinformatics tools, including SIFT, PolyPhen‐2, CADD and Mutation Taster (Table S1, Supporting Information).

To further clarify the effects of this missense variant on HSF5 expression, we separately transfected eukaryotic expression plasmids containing wild‐type (WT)‐*HSF5* and mutant (Mut)‐*HSF5* ^p. R196C^ into HEK293T cells. Western blotting revealed almost no HSF5 expression in cells transfected with a Mut‐*HSF5* ^p.R196C^ plasmid compared to cells transfected with WT‐*HSF5* (Figure S1A, Supporting Information). Moreover, immunofluorescence staining of HSF5 revealed that HSF5 was primarily present in the spermatocytes of testicular biopsy tissues from controls with normal spermatogenesis (Figure S1B, Supporting Information). However, in the two affected individuals, the HSF5 signal was almost completely absent (Figure S1B, Supporting Information). We thus speculated that this *HSF5* homozygous missense mutation leads to a lack of protein expression, which might be responsible for the infertility phenotype of the two siblings.

### 2.2 Meiotic Arrest in Patients Harboring the *HSF5* Variant

To characterize the nature of the spermatogenic arrest in our patients, we performed hematoxylin and eosin (H&E) staining of testis biopsy samples from the patient and controls. In the control testis sample, the spermatogenic epithelium was complete and well arranged, with germ cells at different stages from the basement membrane to the lumen (Figure 1C). In contrast, the patient samples showed disordered and sparse germ cells, including spermatogonia and spermatocytes, and spermatids were rarely observed (Figure 1C). Moreover, a terminal deoxynucleotidyl transferase dUTP nick end labeling (TUNEL) assay showed a significant increase in the number of apoptotic cells in testis sections from patients compared to those from controls (Figure 1D). These results suggested that this human *HSF5* variant might impair spermatogenesis by blocking spermatocyte meiosis, resulting in early maturation arrest and apoptosis of spermatogenic cells during spermatogenesis.

### 2.3 Characterization of the HSF5 Expression Pattern in Mouse Testes

Notably, HSF5 is conserved among mammalian species (Figure S2A, Supporting Information), and its expression in humans is restricted to the testis (Figure S2B, Supporting Information) (https://www.ncbi.nlm.nih.gov/homologene; https://www.proteinatlas.org). Our quantitative PCR (qPCR) assays using various mouse tissues revealed the preferential expression of *Hsf5* in the testes of adult mice (Figure S2C, Supporting Information).

**Figure 2:**
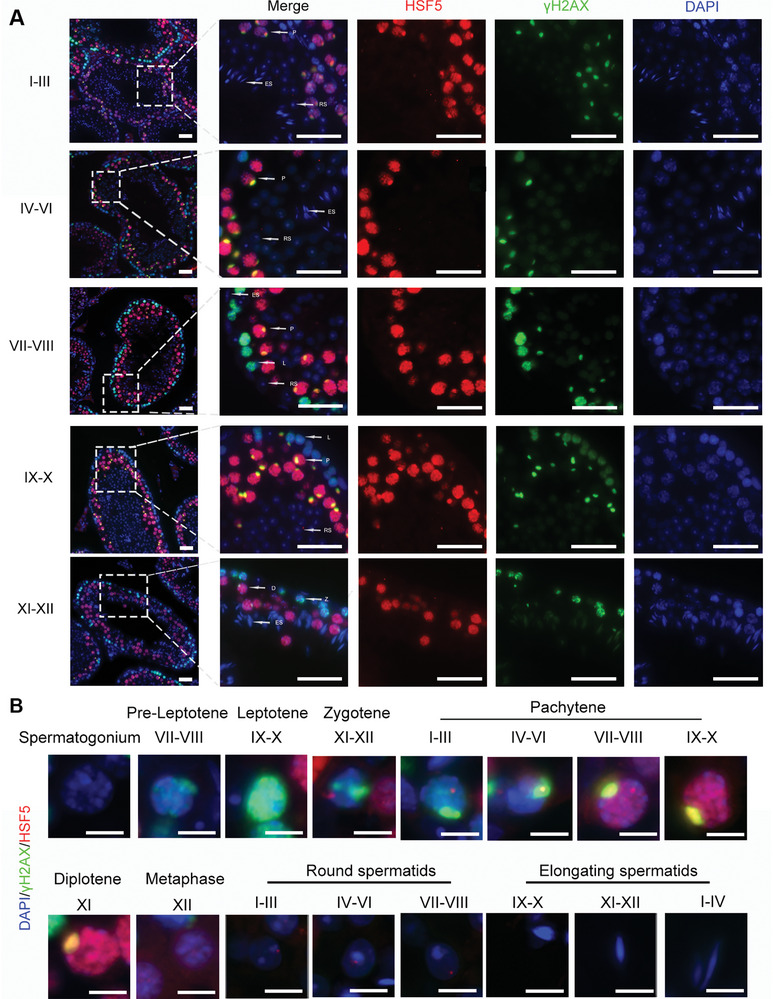
HSF5 shows a dynamic expression pattern during spermatogenesis. A) Immunofluorescence staining for HSF5 and γH2AX in testis sections from 8‐week‐old mice. L, leptotene; Z, zygotene; P, pachytene; D, diplotene; RS, round spermatid; ES, elongating/elongated spermatid (Blue, DAPI; red, HSF5; green, γH2AX; scale bars, 50 µm). B) Immunofluorescence staining of HSF5 and γH2AX in different types of spermatogenic cells. I‐XII, the stages of the spermatogenic epithelial cycle (Blue, DAPI; red, HSF5; green, γH2AX; Scale bars, 10 µm).

To determine the temporal expression profile of HSF5 during spermatogenesis, we performed immunofluorescence staining of HSF5 and γH2AX in mouse testes. During spermatogenesis, γH2AX accumulates at DSB sites during leptotene and zygotene and is then restricted to the XY body during pachytene and diplotene; therefore, γH2AX can be used as a marker to distinguish spermatocytes at different stages.^[^ ^15^ ^]^ Notably, HSF5 was primarily detected in pachytene and diplotene spermatocytes, which were distinguished by intensive γH2AX staining (**Figure** **2**A). Specifically, HSF5 first appeared in zygotene spermatocytes and gradually increased in intensity in pachytene spermatocytes, peaking in diplotene spermatocytes (Figure 2B). Subsequently, HSF5 expression gradually decreased from metaphase spermatocytes to round spermatids and disappeared in elongating spermatids (Figure 2B). Taken together, these data suggested that HSF5 may play a crucial role in spermatogenesis, especially as a key factor in spermatocyte differentiation.

### 2.4 HSF5 is Required for Meiosis and Fertility in Male Mice

To determine whether HSF5 is necessary for fecundity in mammals, we used CRISPR‒Cas9 to generate mice harboring the orthologous variant of *HSF5* ^R196C^ detected in humans to confirm the negative effect of this variant. Unfortunately, due to the complex sequence near this mutated site, the sgRNA designed for this site had low specificity and efficiency, and the generation of *Hsf5* mutant knock‐in mice failed. Instead, since the *HSF5* homozygous mutation caused the absence of HSF5 expression, we generated *Hsf5* knockout (KO) mice to determine the role of HSF5 in spermatogenesis (Figure S3A, Supporting Information). Sanger sequencing, qPCR, western blotting and immunofluorescence staining were performed to confirm that *Hsf5* KO mice were successfully established and that HSF5 expression was absent in the testes of *Hsf5* KO mice (Figure S3B–E, Supporting Information). The *Hsf5* KO mice were viable and appeared to be healthy, with no apparent morphological defects in the reproductive organs of either sex (Figure S3F, Supporting Information). Mating tests revealed that the *Hsf5* KO female mice were fertile, and H&E staining of the ovarian tissue further demonstrated normal oocyte development (Figure S4A,B, Supporting Information). However, repeated breeding of *Hsf5* KO males with WT females failed to yield any births (Figure S4A, Supporting Information). Compared with those of WT testes, the testes of *Hsf5* KO mice were clearly smaller, weighing only 40% that of WT testes (0.0844 ± 0.0043 g vs 0.0352 ± 0.005 g per pair; *P* = 0.0003) (Figure S3G, Supporting Information). The epididymis of *Hsf5* KO mice was also lighter than that of WT mice (0.020 ± 0.002 g versus 0.027 ± 0.001 g per pair; *P* = 0.0063) (Figure S3G, Supporting Information). Moreover, no spermatozoa were collected from the cauda epididymis of *Hsf5* KO mice (Figure S4C, Supporting Information).

**Figure 3:**
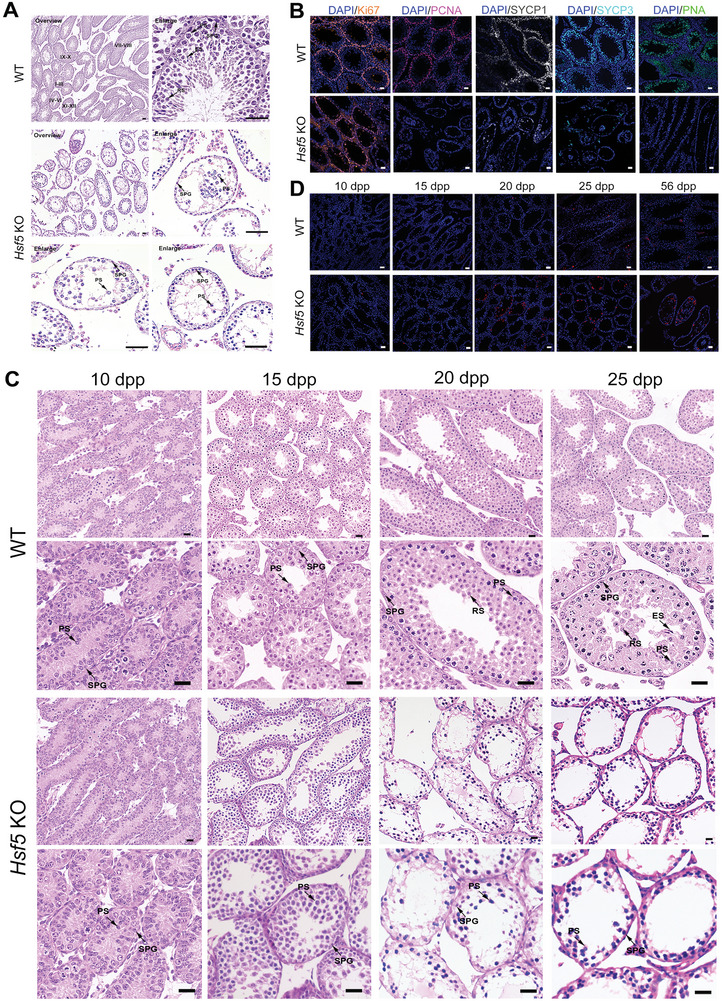
*Hsf5* KO male mice exhibit infertility. A) Sections of testes from 8‐week‐old *Hsf5* KO mice showing a decrease in spermatocytes and an absence of round and elongating/elongated spermatids compared with testes from WT mice. SPG, spermatogonium; PS, primary spermatocyte; RS, round spermatid; ES, elongating/elongated spermatid (N = 3 biologically independent WT mice and KO mice; scale bars, 125 µm). B) Immunofluorescence staining of spermatogenic cell markers in testes from WT and *Hsf5* KO mice (N = 3 biologically independent WT mice and KO mice; blue, DAPI; orange, Ki67; pink, PCNA; grey, SYCP1; sky blue, SYCP3; green, PNA; scale bars, 125 µm). C) Histological analysis of testes from *Hsf5* KO mice of different ages. H&E staining revealed the absence of RS and ES in *Hsf5* KO testes at 20 dpp. SPG, spermatogonium; PS, primary spermatocyte; RS, round spermatid; ES, elongating/elongated spermatid (N = 3 biologically independent WT mice and KO mice; scale bars, 40 µm). D) TUNEL staining of apoptotic germ cells in WT and *Hsf5* KO testis sections of different ages (N = 3 biologically independent WT mice and KO mice; blue, DAPI; red, TUNEL; scale bars, 125 µm).

To elucidate the etiology of infertility in *Hsf5* KO males, we examined the histology of *Hsf5* KO testes by H&E staining. In 8‐week‐old WT testes, normal proliferation and spermatogenesis occurred, and germ cells at different stages were observed (**Figure** **3**A). In contrast, seminiferous tubules in the testes of 8‐week‐old *Hsf5* KO mice had obviously small diameters and were nearly all depleted, exhibiting sparse and disorganized spermatocytes in the tubules (Figure 3A). To further confirm the impairment of spermatogenesis in *Hsf5* KO mice, we examined the expression of markers for different kinds of germ cells. Immunofluorescence staining for Ki67 (a marker of spermatogonia) did not significantly differ between the testes of *Hsf5* KO mice and those of WT mice, but staining for the spermatocyte markers PCNA, SYCP1 and SYCP3 decreased in *Hsf5* KO mice, and staining for the spermatid acrosome marker PNA was absent from the *Hsf5* KO testes (Figure 3B). These results suggested that HSF5 deficiency impairs normal spermatogenesis by arresting cells in the spermatocyte stage, resulting in the failure of mature spermatozoa formation.

Since the first meiotic wave in mouse spermatogenesis begins ≈10 days postpartum (dpp), when preleptotene spermatocytes appear, we compared histomorphological sections of the testes of *Hsf5* KO and WT mice at 10, 15, 20, and 25 dpp (Figure 3C). There were no apparent morphological differences between the WT and *Hsf5* KO testis sections at 10 or 15 dpp, and both spermatogonia and spermatocytes were observed (Figure 3C). A marked discrepancy in the testes of WT and *Hsf5* KO mice was observed at 20 dpp, a significant period in which late pachytene spermatocytes appeared (Figure 3C). Intriguingly, at 20 dpp, round spermatids were visible in WT testes, whereas only spermatogonia and primary spermatocytes were observed in *Hsf5* KO mouse testes (Figure 3C). Moreover, at 25 dpp, the seminiferous tubules of *Hsf5* KO mice did not contain spermatocytes with enlarged nuclei or loose chromosome aggregation (Figure 3C); rather, many highly condensed nuclei, possibly representing apoptotic cells, were observed from 20 to 25 dpp (Figure 3C). Subsequently, TUNEL assays confirmed the elevated numbers of apoptotic germ cells in the testes of *Hsf5* KO mice compared with WT mice at 20 dpp, whereas there was no marked difference at 10 dpp or 15 dpp (Figure 3D), indicating an increase in the apoptosis of late spermatocytes. In summary, knocking out *Hsf5* impeded meiosis in late spermatocytes, which might facilitate apoptosis.

### 2.5 HSF5 Deficiency Induces Primary Spermatocyte Arrest at Late Pachytene

**Figure 4:**
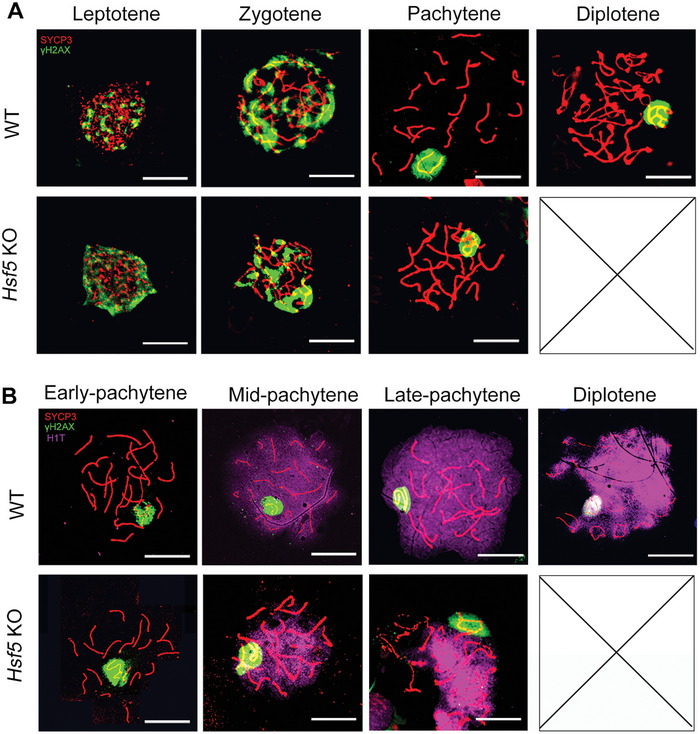
HSF5 is required for meiotic progression beyond the pachytene stage. A) Immunofluorescence staining for γH2AX and SYCP3 in surface‐spread spermatocytes from 8‐week‐old WT and *Hsf5* KO mice. No diplotene spermatocytes were detected in *Hsf5* KO mice (N = 3 biologically independent WT mice and KO mice; green, γH2AX; red, SYCP3; scale bars, 10 µm). B) Immunofluorescence staining for SYCP3, H1t, and γH2AX in spread spermatocytes from 8‐week‐old WT and *Hsf5* KO mice and *Hsf5* KO spermatocytes arrested at late pachytene (N = 3 biologically independent WT mice and KO mice; green, γH2AX; red, SYCP3; purple, H1t; scale bars, 10 µm).

To further confirm the stage at which primary spermatocyte arrest occurs, we examined meiotic chromosome spreads with coimmunostaining for SYCP3 and γH2AX. The staining results showed that leptotene‐, zygotene‐, and pachytene‐stage spermatocytes were present in *Hsf5* KO, but no diplotene‐stage spermatocytes were observed (**Figure** **4A**; Figure S5, Supporting Information), indicating that *Hsf5* KO spermatocytes arrested before diplonema. To further investigate the specific pachytene stage at which arrest of *Hsf5* KO spermatocytes occurred, we performed immunostaining for histone H1t, which begins to be expressed at the mid‐pachytene stage and then gradually increases in intensity until diplotene termination in spermatocytes. Two classes of H1t‐positive spermatocytes were detected in *Hsf5* KO mice: those exhibiting a mild H1t signal were classified as mid‐pachytene spermatocytes, while those stained with a strong H1t signal and exhibiting fractured and fragmented chromosomes were categorized as late‐pachytene spermatocytes (Figure 4B; Figure S5, Supporting Information). Therefore, meiosis in *Hsf5* KO spermatocytes arrested at the late pachytene stage and could not progress through the pachytene checkpoint.

### 2.6 HSF5 is Indispensable for the Normal Progression of Meiotic Recombination, Synapsis, Crossover Formation and MSCI

**Figure 5:**
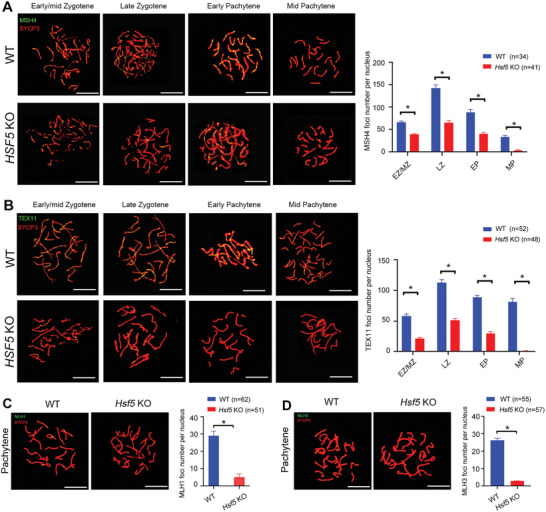
HSF5 is related to crossover formation and late recombination during meiosis. A) Immunofluorescence staining of MSH4 and SYCP3 in spread spermatocytes at successive prophase stages from 8‐week‐old WT and *Hsf5* KO mice, including early/mid‐zygotene, late zygotene, early pachytene and mid‐pachytene stages. The number of MSH4 foci decreased in *Hsf5* KO spermatocytes compared to WT spermatocytes in these stages. Quantification of MSH4 foci per nucleus at the indicated substages (N = 3 biologically independent WT mice and KO mice; n, the total number of nuclei analyzed; two‐sided Student's *t* test; *P < 0.05; error bars, mean ± SEM; green, MSH4; red, SYCP3; scale bars, 10 µm). B) Immunofluorescence staining showing the expression of TEX11 and SYCP3 in spread spermatocytes at successive prophase stages from 8‐week‐old WT and *Hsf5* KO mice. TEX11 foci were more readily detected in *Hsf5* KO spermatocytes than in WT spermatocytes. Quantification of TEX11 foci per nucleus at the indicated substages (N = 3 biologically independent WT mice and KO mice; n, the total number of nuclei analyzed; two‐sided Student's *t* test; *P < 0.05; error bars, mean ± SEM; green, TEX11; red, SYCP3; scale bars, 10 µm). C) Representative images of pachynema spermatocytes stained for MLH1 and SYCP3 from 8‐week‐old WT and *Hsf5* KO mice. Almost no MLH1 signal was detected in *Hsf5* KO spermatocytes compared to WT spermatocytes. Quantification of MLH1 foci per nucleus in the pachynema spermatocytes (N = 3 biologically independent WT mice and KO mice; n, the total number of nuclei analyzed; two‐sided Student's *t* test; *P < 0.05; error bars, mean ± SEM; green, MLH1; red, SYCP3; scale bars, 10 µm). D) Spread spermatocytes from 8‐week‐old WT and *Hsf5* KO mice were stained for MLH3 and SYCP3. The numbers of MLH3 foci were sharply reduced in *Hsf5* KO spermatocytes. Quantification of MLH3 foci per nucleus in the pachynema spermatocytes (N = 3 biologically independent WT mice and KO mice; n, the total number of nuclei analyzed; two‐sided Student's *t* test; *P < 0.05; error bars, mean ± SEM; green, MLH3; red, SYCP3; scale bars, 10 µm).

Initiation of programmed DSBs appeared to be normal in *Hsf5* KO spermatocytes, as shown by γH2AX staining (Figure 4A). The signals of two key early‐stage recombination markers, RAD51 and RPA2,^[^ ^16^ ^]^ were also similar in the WT and *Hsf5* KO spermatocytes (Figure S6A,B, Supporting Information). Interestingly, the signals of MSH4 and TEX11, two well‐known factors involved in later steps of the recombination process,^[^ ^16^ ^]^ were significantly decreased in *Hsf5* KO pachytene spermatocytes compared with WT pachytene spermatocytes, suggesting instability of the late stage of recombination in *Hsf5* KO spermatocytes (**Figure** **5**A,B). These findings suggested that HSF5 is essential for late recombination during meiosis.

We further costained spermatocytes for the crossover‐specific marker MLH1^[^ ^17^ ^]^ and SYCP3 to investigate whether crossover formation was affected in *Hsf5* KO spermatocytes. Notably, the average number of MLH1 foci per nucleus was 28.3 ± 3.2 in WT pachytene spermatocytes but only 11.2 ± 1.5 in *Hsf5* KO spermatocytes (Figure 5C). Similarly, foci of MLH3, another crossover marker, were nearly absent in *Hsf5* KO spermatocytes (Figure 5D), indicating a disruption of crossover.

**Figure 6:**
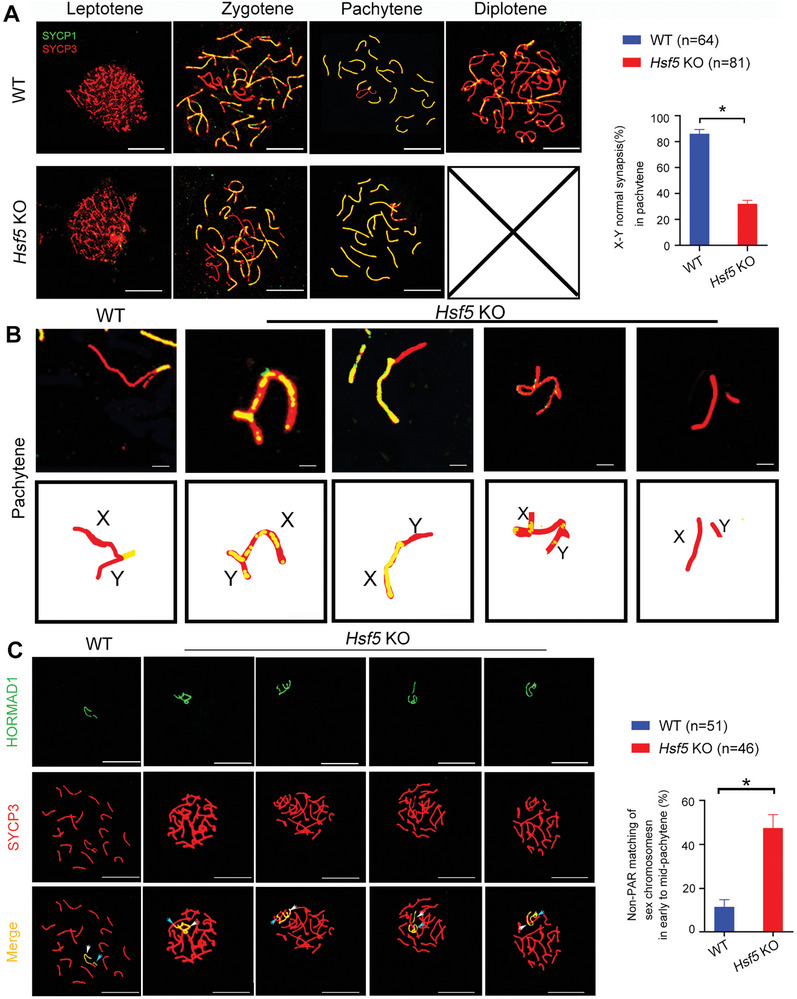
*Hsf5* KO male mice exhibit abnormalities in sex chromosome synapses. A) Synapsis analyses of spermatocytes from WT and *Hsf5* KO mice with SYCP3 and SYCP1 by immunostaining surface‐spread spermatocytes. *Hsf5* KO mice exhibited abnormal sex chromosome synapsis. Quantification of X‐Y normal chromosome synapsis per nucleus in pachytene stage (N = 3 biologically independent WT mice and KO mice; n, the total number of nuclei analyzed; two‐sided Student's *t* test; *P < 0.05; error bars, mean ± SEM; green, SYCP1; red, SYCP3; scale bars, 10 µm). B) The magnified panels showed abnormally synapsed sex chromosomes (Green, SYCP1; red, SYCP3; scale bars, 10 µm). C) Chromosome spreads of WT and *Hsf5* KO spermatocytes were stained for SYCP3 and HORMAD1. Quantification of non‐PAR X‐Y matching per nucleus in pachynema spermatocytes (N = 3 biologically independent WT mice and KO mice; n, the total number of nuclei analyzed; two‐sided Student's *t* test; *P < 0.05; error bars, mean ± SEM; green, HORMAD1; red, SYCP3; scale bars, 10 µm). The white arrow indicates the X chromosome, and the blue arrow indicates the Y chromosome.

Given that homologous recombination and synapsis are interdependent and that recombination promotes synapsis,^[^ ^18^ ^]^ we further coimmunostained *Hsf5* KO spermatocytes for SYCP3 and SYCP1. In WT spermatocytes, SYCP1 began to colocalize with SYCP3 when homologous chromosomes became paired and initiated synapsis in the zygotene stage and became completely colocalized at the pachytene stage, except in the non‐pseudoautosomal region (non‐PAR, the non‐synapsed sex chromosomal region) of the X and Y chromosomes (**Figure** **6**A). However, incorrectly synapsed sex chromosomes were detected in *Hsf5*‐deficient spermatocytes (Figure 6A). The percentage of normal sex chromosome synapsis in *Hsf5* KO spermatocytes was significantly reduced (Figure 6A). Specifically, the staining of SYCP1 on the X and Y chromosomes revealed no synapsis in the PAR, or inappropriate synapsis in the non‐PAR (Figure 6B). The above observations were further substantiated by staining for TEX12, another structural component of the synaptonemal complex (SC) (Figure S7, Supporting Information). We further assessed staining for HORMAD1, which is localized along the unsynapsed chromosome axis in pachynema.^[^ ^19^ ^]^ In WT mice, the HORMAD1 signal was observed only at unsynapsed sex chromosomal regions in the pachytene stage (Figure 6C). However, in *Hsf5* KO spermatocytes, HORMAD1 staining was anomalously detected in the PARs of sex chromosomes (Figure 6C). These findings showed that the absence of HSF5 might cause abnormal synapsis during meiosis.

Asynapsis in the early pachytene stage can result in meiotic silencing of unsynapsed chromatin (MSUC), involving the recruitment of proteins such as BRCA1 and ATR to synapsed axes and the accumulation of γH2AX at associated chromatin loops, further activating checkpoints and apoptosis in the pachynema and ultimately leading to sterility.^[^ ^20^, ^21^ ^]^ MSCI, a specialized form of MSUC, involves transcriptional silencing of the unsynapsed region of the X and Y chromosomes, which normally occurs at the pachytene stage.^[^ ^22^ ^]^ To further confirm the disruption of MSCI by the unsynapsed PAR of the X and Y chromosomes in KO spermatocytes, as observed above, we first examined the expression of p‐RNAP, which reflects the transcriptional activity of the sex chromosomes. As reported previously, p‐RNAP was detected on the autosomes of all bivalents, except for the X and Y chromosomes, which remained unmarked at the pachytene stage in WT spermatocytes (Figure S8A, Supporting Information). However, positive staining signals for p‐RNAP on the X and Y chromosomes were substantially increased in *Hsf5* KO spermatocytes (Figure S8A, Supporting Information). Similar to p‐RNAP, H3k4me3, an epigenetic modification enriched in transcription activation regions, was largely excluded from the X and Y chromosomes of WT spermatocytes at the pachytene stage (Figure S8B, Supporting Information). However, in *Hsf5*‐deficient pachytene spermatocytes, the X and Y chromosomes exhibited obvious H3k4me3 staining along their entire lengths (Figure S8B, Supporting Information). Moreover, pachytene spermatocytes were isolated from WT and *Hsf5* KO testes, and the expression of several genes encoded on the autosomes (*Zp2*, *Cep128*, and *Qrich2*) and sex chromosomes (*Slc25a5*, *Usp26*, *Usp9y*, *Rmby*, *Sry*, and *Zfy2*) was measured. The results revealed that the transcription of sex chromosome genes was obviously greater in *Hsf5* KO pachytene spermatocytes than in WT spermatocytes, while the expression of autosomal genes was not significantly different between WT and *Hsf5* KO mice (Figure S8C, Supporting Information). These results collectively showed that HSF5 is indispensable for MSCI.

### 2.7 HSF5 Binds Predominantly to the Promoters of Key Meiosis‐Related Genes

**Figure 7:**
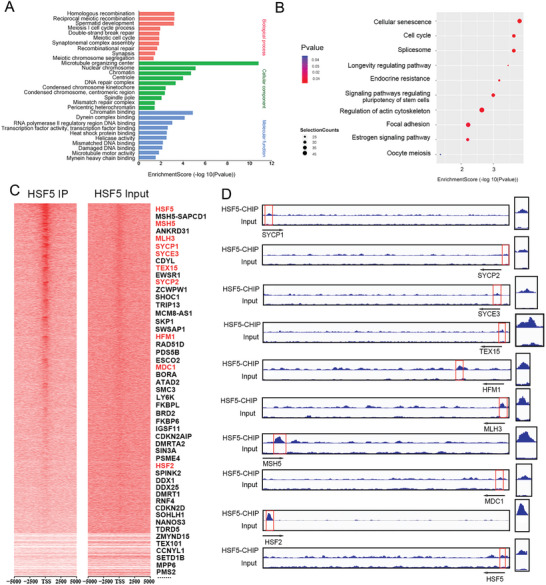
HSF5 occupies the promoters of meiosis‐related genes. A) GO analysis showing the top 10 enriched biological processes, cellular components and molecular functions. B) KEGG pathway analysis of the genes with HSF5 binding peaks in their promoter regions. C) Heatmaps of HSF5 ChIP‐seq data and input signals. D) HSF5 occupancy at the promoters of representative meiosis‐related genes.

Given that HSF5 is a transcription factor (TF), we speculated that HSF5 may bind to the promoters of specific target genes involved in spermatogenesis. The currently available HSF5 antibody‐targeting ChIP experiments have higher specificity for the human protein than for the mouse protein; thus, we performed ChIP‐seq on human testis samples from obstructive azoospermia patients. A total of 9601 binding sites were identified, 12.58% of which resided within 2000 bp of promoter regions in the human genome (Figure S9A, Supporting Information). We then analyzed the top 5 motifs for both known and de novo motifs, respectively (Figure S9B, Supporting Information). HSF5 commonly bound to 3300 genes, and most of the binding sites were within ± 1 kb of the transcription start site (TSS) (Figure S9C, Supporting Information). Additionally, HSF5 exhibited the highest binding strength to chromosomes 1, 2, and 19, and there were no peaks on the Y chromosome (Figure S9D, Supporting Information). Remarkably, GO analysis of HSF5‐ChIP targets revealed enrichment at genes associated with meiotic processes (**Figure** **7**A). KEGG analysis also revealed enrichment in signaling pathways related to reproductive processes (Figure 7B). Among the target genes involved in meiosis, we confirmed that eight genes essential for meiotic dynamics were directly activated by HSF5: the SC component genes *SYCP1*, *SYCP2*, and *SYCE3*; the recombination‐related gene *TEX15*; the crossover‐formation genes *HFM1*, *MSH5* and *MLH3;* and the MSCI gene *MDC1* (Figure 7C,D). Intriguingly, the promoters of *HSF5* and *HSF2* were also identified, suggesting that HSF5 might be important for *HSFs* expression (Figure 7C,D).

**Figure 8:**
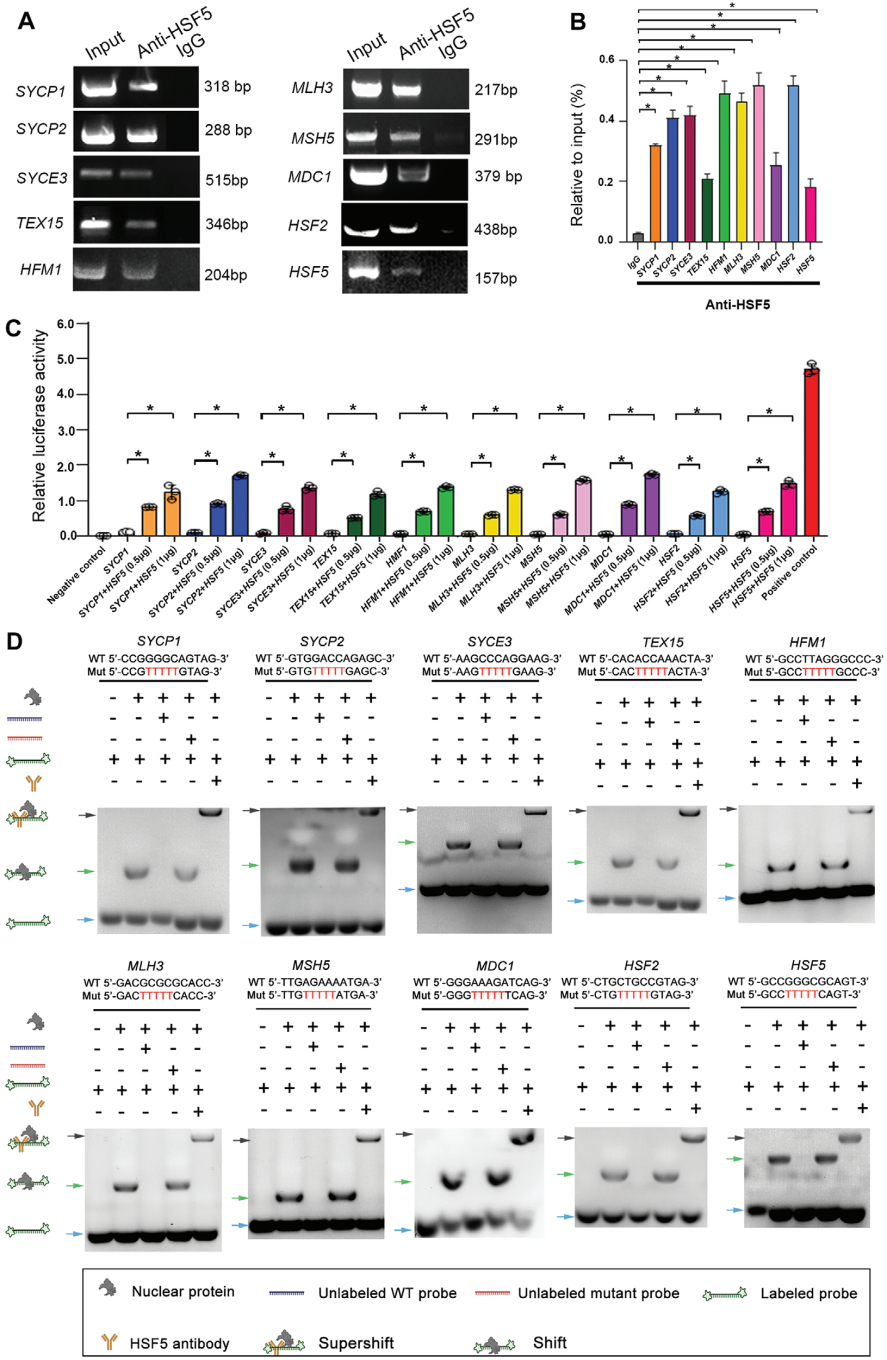
Requirement of DNA binding for HSF5 to regulate meiosis‐related gene expression. A) ChIP‒PCR was used to detect HSF5‐occupied sites in the promoters of target genes in human testicular tissues. Input DNA and mouse IgG pulldowns were used as positive controls and negative controls, respectively. Three independent experiments were performed. B) ChIP‒qPCR results showing increased binding of HSF5 to gene promoter regions in the group treated with the HSF5 antibody compared to that in the group treated with IgG. Three independent experiments were performed. (Two‐sided Student's *t* test; *P < 0.05; error bars, mean ± SEM). C) HSF5 enhanced the transcriptional activity of target genes, as shown by luciferase reporter assays. Three independent experiments were performed. (Two‐sided Student's *t* test; error bars, *P < 0.05; error bars, mean ± SEM). D) HSF5 binding to a specific sequence in the proximal promoter of target genes, as determined by EMSA. Three independent experiments were performed.

We next performed ChIP‒PCR and confirmed that HSF5 could bind to the promoter regions of *SYCP1*, *SYCP2*, *SYCE3*, *TEX15*, *MDC1*, *HFM1*, *MSH5*, *MLH3*, *HSF2* and *HSF5* (**Figure** **8**A). Similarly, ChIP‒qPCR results showed that the binding of HSF5 to the promoter fragments of *SYCP1*, *SYCP2*, *SYCE3*, *TEX15*, *MDC1*, *HFM1*, *MSH5*, *MLH3*, *HSF2* and *HSF5* increased by approximately 10‐fold, 12‐fold, 12‐fold, 7‐fold, 8‐fold, 16‐fold, 15‐fold, 17‐fold, 15‐fold and 6‐fold, respectively (Figure 8B). We performed a dual‐luciferase reporter assay and observed that the luciferase activity of constructs bearing the promoter fragments of *SYCP1*, *SYCP2*, *SYCE3*, *TEX15*, *MDC1*, *HFM1*, *MSH5*, *MLH3*, *HSF2* and *HSF5* increased with increasing overexpression of HSF5 in HEK293T cells (Figure 8C). To validate the direct binding of HSF5 to the promoters of *SYCP1*, *SYCP2*, *SYCE3*, *TEX15*, *MDC1*, *HFM1*, *MSH5*, *MLH3*, *HSF2* and *HSF5*, we performed electrophoretic mobility shift assay (EMSA) experiments to investigate the binding activity of HSF5 from nuclear extracts to the candidate nucleotide sequences of these genes. In ChIP‐seq, biotinylated double‐stranded oligonucleotides from target gene promoters were synthesized as labeled probes and tested for their ability to bind HSF5. Then, HEK293T cells were transfected with the Flag‐WT‐*HSF5* plasmid, and nuclear extracts from these cells were used to analyze the DNA binding activity of HSF5. The ideal probes containing the promoter sequences of *SYCP1*, *SYCP2*, *SYCE3*, *TEX15*, *MDC1*, *HFM1*, *MSH5*, *MLH3*, *HSF2* and *HSF5* are shown in Figure 8D. The results showed that the HSF5 nuclear extracts could bind to labeled WT probes containing the *SYCP1*, *SYCP2*, *SYCE3*, *TEX15*, *MDC1*, *HFM1*, *MSH5*, *MLH3*, *HSF2* and *HSF5* promoters. To confirm that the binding between HSF5 and the above probes was specific, we added synthesized mutant probes and excess unlabeled WT probes. As expected, we observed a significant decrease in or complete disappearance of HSF5 target binding capacity when extracts were tested with mutant probes or unlabeled WT probes (Figure 8D). Moreover, the protein–probe complex was recognized and bound by the anti‐HSF5 antibody, confirming the specificity of the antibody (Figure 8D). These data suggested that HSF5 binds directly to the promoters of key meiosis‐related genes and then regulates their transcription during spermatogenesis.

### 2.8 Massive Alterations in the Transcription of Meiosis‐Related Genes in *Hsf5* KO Juvenile Mice

**Figure 9:**
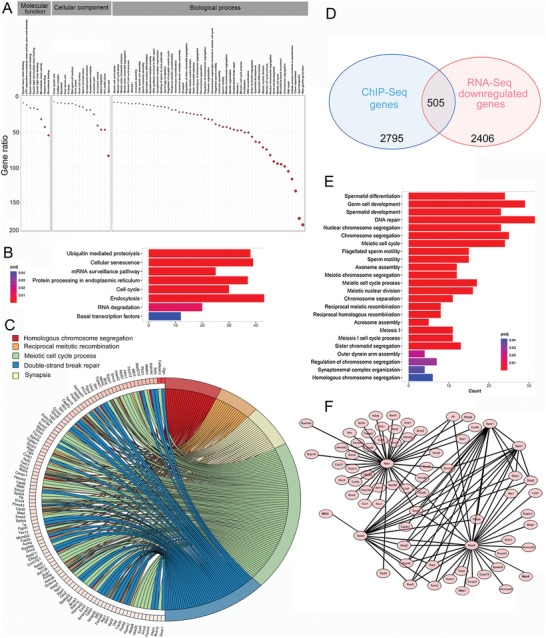
Loss of *Hsf5* leads to impaired spermatogenesis according to RNA‐seq analysis. A) GO analysis showed that the genes with diminished expression in the testes of *Hsf5* KO mice were mostly involved in spermatogenesis. B) KEGG analysis indicated enrichment of the pathway involved in transcriptional regulation. C) Connection network of downregulated genes participating in meiotic progression in *Hsf5* KO mice. D) Overlap between downregulated genes in *Hsf5* KO mice and HSF5‐bound genes. E) GO analysis of the overlapping genes. F) PPI analysis showing the interaction network formed by *Sycp1*, *Sycp2*, *Syce3*, *Tex15*, *Mdc1*, *Hfm1*, *Mlh3*, and *Msh5*.

We next conducted RNA‐seq analysis to identify *Hsf5*‐regulated genes that were differentially expressed in the testes of *Hsf5* KO and WT mice. We performed this analysis at approximately 20 dpp because WT juvenile testes at 20 dpp are enriched in pachytene spermatocytes. Differential expression analysis revealed that 2911 and 1885 genes were downregulated and upregulated, respectively, when *Hsf5* was knocked out (Figure S10A, Supporting Information). We found that 94 downregulated genes were specifically linked to meiosis (Figure S10B, Supporting Information). GO analysis revealed that almost all the differentially expressed genes were involved in spermatogenesis, especially the meiotic process, including meiosis I cell cycle process, SC organization, and DNA recombination (**Figure** **9**A). KEGG analysis revealed that the downregulated genes were enriched in cell cycle signaling and basal TFs, reflecting the main biological function of HSF5 (Figure 9B). We further found extensive crosstalk among the downregulated genes involved in reciprocal meiotic recombination, synapsis, DSB repair, and homologous chromosome segregation (Figure 9C).

**Figure 10:**
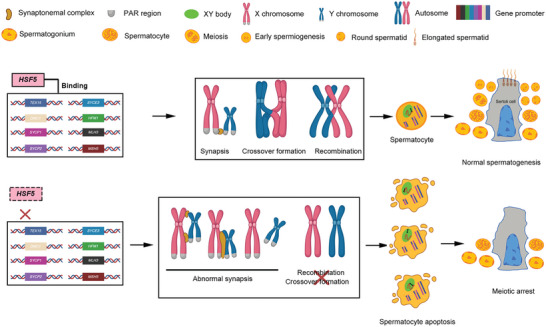
Working model of the transcriptional regulation of HSF5 during male meiosis. HSF5 functions as a transcription factor that binds to the promoters of meiosis‐related genes, including synapsis‐related genes (e.g., *SYCP1*, *SYCP2* and *SYCE3*), the recombination‐related gene *TEX15*, crossover formation genes (e.g., *HFM1*, *MLH3* and *MSH5*) and the MSCI gene *MDC1*, to maintain their expression; deficiency in HSF5 causes aberrant meiotic sex chromosome synapsis, recombination, crossover formation, and MSCI, which leads to spermatocyte apoptosis and meiotic arrest at the late pachytene stage. Dashed boxes indicate compromised function. Created with BioRender.com.

To determine whether the transcriptional changes in *Hsf5* KO testes were directly regulated by HSF5, we tested whether there was significant overlap between the differentially expressed genes in *Hsf5* KO testes and HSF5‐bound genes. As expected, HSF5‐bound genes were significantly enriched among the genes downregulated in *Hsf5* KO testes (Figure 9D). The overlapping genes were mainly involved in the key biological events of meiosis, according to GO analysis (Figure 9E). The downregulation of *Sycp1*, *Sycp2*, *Syce3*, *Tex15*, *Mdc1*, *Hfm1*, *Msh5*, *Mlh3*, *Hsf2* and *Hsf5* in KO mice compared to WT mice was particularly evident and was further validated by qPCR (Figure S11, Supporting Information). PPI analysis revealed that almost all of the interactors in the network formed by *Sycp1*, *Sycp2*, *Syce3*, *Tex15*, *Hfm1*, *Msh5*, *Mlh3*, and *Mdc1* were related to meiosis (Figure 9F). Overall, our findings demonstrated that HSF5 binds to the promoters of meiosis‐related genes, including *SYCP1*, *SYCP2*, *SYCE3*, *TEX15*, *MDC1*, *HFM1*, *MSH5*, and *MLH3*, the deficiency of which leads to defects in meiotic recombination, synapsis, crossover and the MSCI, resulting in meiosis arrest at the late pachytene stage (**Figure** **10**).

### 2.9 Rescue of Infertility in *Hsf5* KO Mice

Adeno‐associated virus (AAV) allows efficient germline and niche manipulation as it penetrates the basement membrane of seminiferous tubules, providing a promising strategy for the development of gene therapies for reproductive defects.^[^ ^23^ ^]^ Currently, AAV1, AAV8, and AAV9 are applied and are known to effectively penetrate the blood‒testis barrier (BTB).^[^ ^23^, ^24^ ^]^ We screened different titers of the three AAVs and found that 8 × 10^10^ genomic copies (gc) of AAV9 exhibited the highest efficiency in mouse testes (Figure S12, Supporting Information).

**Figure 11:**
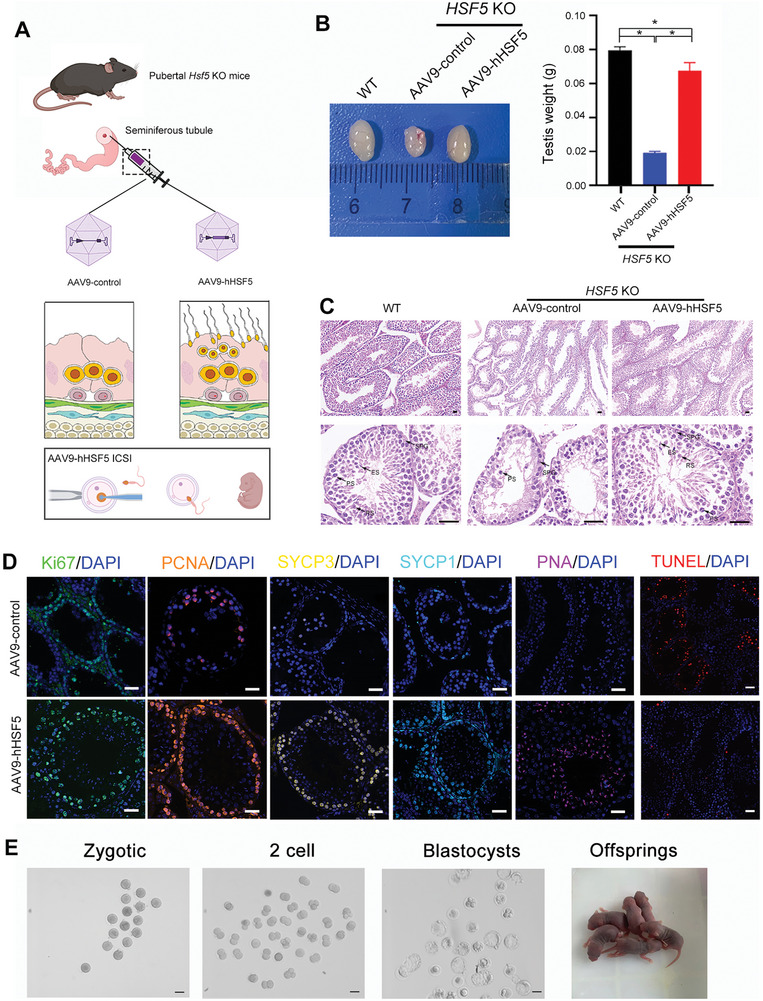
AAV9‐hHSF5 rescues spermatogenesis in *Hsf5* KO mice. A) Working model for AAV‐hHSF5‐mediated recovery of spermatogenesis. Created with BioRender.com. B) Macroscopic images and weights of WT and *Hsf5* KO mouse testes after microinjection of AAV9‐control (WT and *Hsf5* KO respectively) or AAV9‐hHSF5 (*Hsf5* KO) (N = 3 biologically independent WT mice and KO mice; two‐sided Student's *t* test; *P < 0.05; error bars, mean ± SEM). C) Histological analysis of testis sections collected from WT and *Hsf5* KO mice injected with AAV9‐control or AAV9‐hHSF5. SPG, spermatogonium; PS, primary spermatocyte; RS, round spermatid; ES, elongating/elongated spermatid (N = 3 biologically independent WT mice and KO mice; scale bars, 125 µm). D) Immunofluorescence staining of signals marking different germ cells and apoptotic cells (N = 3 biologically independent KO mice; blue, DAPI; green, Ki67; orange, PCNA; yellow, SYCP3; sky blue, SYCP1; pink, PNA; red, TUNEL; scale bars, 125 µm). E) Images of one‐cell zygotes, two‐cell embryos, and blastocysts and offspring after ICSI using sperm from AAV9‐hHSF5‐injected *Hsf5* KO male mice (N = 3 biologically independent KO mice; scale bars, 100 µm).

To investigate whether the meiotic arrest phenotype of *Hsf5* KO mice could be rescued by AAV‐based gene therapy, we constructed AAV9‐control‐mScarlet and AAV9‐hHSF5‐P2A‐mScarlet constructs and microinjected the two AAV9s into the seminiferous tubules of‐3‐week‐old male *Hsf5* KO mice, and 3‐week‐old WT male mice microinjected with AAV9‐control as a control group (**Figure** **11**A). To evaluate the gene transfer efficiency, we measured the levels of HSF5 five weeks after AAV9 administration. Immunofluorescence staining revealed that the HSF5 expression in the testes of the KO mice treated with AAV9‐hHSF5 was comparable to that in WT mice, whereas negligible HSF5 was detected in the testes of the KO mice treated with AAV9‐control (Figure S13, Supporting Information).

Furthermore, we assessed whether spermatogenesis was restored in the AAV9‐hHSF5‐rescued mice. Notably, although the testes of *Hsf5* KO mice that received AAV9‐hHSF5 (0.068 ± 0.001 g) were lighter than those of WT mice (0.08 ± 0.002 g), they were significantly heavier than those of the KO mice that received AAV9‐control (0.019±0.001 g) (Figure 11B). Moreover, histological analysis of testes from *Hsf5* KO mice with AAV9‐hHSF5 revealed increased seminiferous tubule width, accompanied by substantial spermatocytes, round spermatids and elongating spermatids (Figure 11C). In contrast, the seminiferous tubule diameters in AAV9‐control‐treated *Hsf5* KO mice remained reduced, and spermatogenesis was arrested at the spermatocyte stage (Figure 11C). Moreover, immunofluorescence staining of various germ cell markers further confirmed the rescue of spermatogenesis by AAV9‐hHSF5 injection (Figure 11D). In addition, few TUNEL‐positive apoptotic cells were detected in the seminiferous tubules of KO mice injected with AAV9‐hHSF5, while many TUNEL‐positive apoptotic cells remained in those of the AAV9‐control group (Figure 11D).

We next investigated whether *Hsf5* KO mice generated via AAV9‐hHSF5 gene therapy could produce offspring through natural mating. Three mature *Hsf5* KO male mice generated from AAV9‐hHSF5 administration were mated with five WT females. Regrettably, none of the AAV9‐hHSF5‐treated *Hsf5* KO mice produced offspring after 3 months of natural mating. We further explored whether offspring could be produced via assisted reproduction with the sperm produced after AAV9‐hHSF5 gene therapy. ICSI was performed using spermatozoa obtained from the caudal epididymis of rescued *Hsf5* KO male mice and oocytes harvested from female WT mice. The fertilization rates, two‐cell rates, and blastocyst rates of the rescued mice were 72.78%, 65.23%, and 41.72%, respectively, and healthy offspring were ultimately obtained (Figure 11E). Thus, AAV9‐hHSF5 treatment restored spermatogenesis in *Hsf5* KO mice and further promoted to produce health offspring (Figure 11A,E). Collectively, our data suggested that the AAV gene therapy strategy could be a promising treatment for meiotic arrest in mice.

## 3 Discussion

As the foundation of sexual reproduction, meiosis is required to ensure genome stability and heritable diversity by generating haploid gametes through the formation of DSBs, homologous pairing, synapsis, interhomologous recombination, and crossover formation.^[^ ^5^ ^]^ Meiotic arrest is a major cause of male infertility, and more than 100 genes have been identified to be essential for meiosis in mice,^[^ ^25^ ^]^ whereas only 21 genes have been strongly associated with meiotic arrest in human infertility patients to date.^[^ ^26^ ^]^ Therefore, it is crucial to explore the pathogenic genes involved in meiotic arrest in humans and further understand the mechanisms underlying their effects on fertility. Importantly, in this study, we identified *HSF5* as a novel factor in spermatocyte meiosis in humans that is involved in regulating the transcription of genes related to key meiotic events, namely, synapsis, recombination, crossover formation and MSCI. Our findings expanded our knowledge of human meiosis and further provided valuable information for the clinical genetic diagnosis and treatment of meiotic arrest patients.

Heat shock transcription factors (HSFs) are well known as transcriptional regulators of genes encoding heat shock proteins and others and have functions in reproduction, the immune response and the aging process.^[^ ^12^ ^]^ The mammalian HSF family includes HSF1, HSF2, HSF3, HSF4, HSF5, HSFY, and HSFX. Previous experiments on the inactivation (knockout) of *Hsf* genes in mice have yielded evidence that two members play prominent roles in spermatogenesis.^[^ ^12^ ^]^ Although they are fertile, *Hsf1* null males produce less sperm than WT mice do, and approximately 40% of their epididymal spermatozoa exhibit abnormal head morphology.^[^ ^27^ ^]^ Defective spermatogenesis was also observed in male mice lacking *Hsf2*, including markedly reduced sperm count and quality leading to a small decrease in fertility.^[^ ^28^, ^29^ ^]^ Recently, another laboratory in parallel reported that HSF5 deficiency resulted in male infertility in mice, which was linked to impaired MSCI.^[^ ^30^ ^]^ However, the relevant evidence needs to be explored further. In our study, we observed similar defects in MSCI in *Hsf5* KO mice. Notably, we detected abnormalities in the synapsis of the sex chromosome PARs, aberrations in late recombination, and deficiencies in crossover formation, resulting in meiosis arrest at late pachynema. Crucially, our ChIP‐seq combined with EMSA indicated that HSF5 binds to the promoters of genes associated with meiosis I, such as *SYCP1*, *SYCP2, SYCE3*, *TEX15*, *HFM1*, *MSH5*, *MLH3*, and *MDC1*, and RNA‐seq analysis indicated that these genes were downregulated in *Hsf5* KO mice. Most importantly, we revealed for the first time that loss‐of‐function mutations in *HSF5* are a genetic cause of meiotic arrest in humans. Overall, our study provides a comprehensive investigation of HSF5 function in mammals and clarifies the mechanism by which HSF5 regulates the meiosis process, shedding light on the essential role of the HSF family in reproduction.

Previous findings have shown that single *Hsf1*‐null male mice exhibit morphological abnormalities in their sperm heads but are fertile.^[^ ^27^ ^]^ Single *Hsf2*‐null male mice also exhibit a small impairment in male fertility and still produce offspring.^[^ ^28^, ^29^ ^]^ Interestingly, simultaneous deficiency of both *Hsf1* and *Hsf2* results in the complete absence of sperm and spermatocyte arrest at the pachytene stage, resulting in complete infertility.^[^ ^29^ ^]^ Therefore, it is speculated that HSF1 and HSF2 might have a compensatory relationship. In the present study, single *Hsf5* KO male mice exhibited complete meiosis arrest, and almost no postmeiotic cells were found in these animals. These findings suggested that HSF1 and HSF2 might have no or a weak compensatory effect for HSF5; that is, the loss of HSF5 alone produced a completely infertile phenotype. Moreover, we demonstrated that HSF5 binds to its own promoter and that of *HSF2*, suggesting that HSF5 is critical for the transcriptional regulation of *HSF2* and itself. Further studies could elucidate the links between HSF family members.

To date, surgical testicular sperm extraction combined with ICSI has been essentially the only approach by which primary meiotic arrest patients can achieve biological offspring. However, this strategy has shown limited success; only 28% of patients can be effectively treated.^[^ ^4^ ^]^ In recent years, gene therapy has been considered a promising potential therapeutic strategy for male infertility caused by meiotic arrest. In previous studies, genes have been successfully transduced into the testis by microinjection of lentiviruses or adenoviruses.^[^ ^23^, ^24^, ^31^, ^32^ ^]^ However, there are still evident limitations, such as the inflammatory reaction caused by adenovirus injection, the decline in long‐term gene expression from the adenovirus, and the unwanted transduction of genes into germ cells by lentivirus.^[^ ^23^, ^24^, ^31^, ^32^, ^33^ ^]^ One candidate gene delivery vector that can overcome these problems is AAVs, which have been applied to treat some genetic diseases, such as hemophilia, retinitis pigmentosa, and deafness.^[^ ^34^, ^35^, ^36^, ^37^ ^]^ However, findings on the use of AAV‐mediated gene delivery for the treatment of male infertility are limited. Watanabe et al. suggested that AAV1/9 can penetrate the BTB and the basement membrane of the seminiferous tubules to transduce both spermatogonial stem cells and Sertoli cells without harmful reactions or AAV integration in offspring.^[^ ^23^ ^]^ Another study identified AAV8 as an efficient vector that drives exogenous *Lhcgr* expression in the Leydig cells of *Lhcgr* KO mice after interstitial injection and revealed substantially restored spermatogenesis in KO mice, which effectively produced fertile offspring via ICSI after rescue.^[^ ^24^ ^]^ In our study, we initially injected AAV serotypes 1, 8, and 9 at doses of 4 × 10^10^ gc, 8 × 10^10^ gc, and 1.6 × 10^11^ gc into each testis of WT mice at 3 weeks and found that AAV9 at 8 × 10^10^ gc exhibited the highest efficiency among the tested serotypes. We then microinjected AAV9‐hHSF5 of 8 × 10^10^ gc into the seminiferous tubules of *Hsf5* KO male mice and found that spermatogenesis was effectively restored without any harmful effects. Although none of the gene‐treated *Hsf5* KO mice produced offspring in 3 months of natural mating, ICSI treatment was performed using spermatozoa obtained from the caudal epididymis of treated *Hsf5* KO mice, and the offspring were successfully born with no AAV integration. Therefore, AAV‐mediated gene therapy might be appropriate for treating meiotic arrest in animals and humans, and gene transduction via AAVs may be useful for studying the mechanisms of spermatogenesis.

Mutations in TF‐encoding genes have been reported to be associated with human infertility, including impaired sex development, primary ovarian insufficiency and nonobstructive azoospermia.^[^ ^38^, ^39^ ^]^ However, strong evidence of a contribution to meiotic arrest has been uncovered for only two TFs.^[^ ^39^ ^]^ SOHLH1 is a germ cell‐specific TF that is required for spermatocyte production and occupies an E‐box‐containing region.^[^ ^40^ ^]^ *Sohlh1* KO mice displayed meiotic defects at the zygotene stage, and dysfunction of SOHLH1 also impaired spermatogenesis in spermatocytes in humans.^[^ ^41^, ^42^ ^]^ MEIOSIN, a germ‐cell‐specific TF that binds to the meiotic gene promoters, plays a pivotal role in activating meiotic process.^[^ ^43^ ^]^ Homozygous *Meiosin* mutant mice both female and male mice were infertile, and presented the meiotic prophase I process disruption.^[^ ^44^ ^]^ Moreover, MEIOSIN deficiency contributes to human primary ovarian insufficiency by impairing meiosis due to inadequate transcriptional activation of meiotic genes.^[^ ^44^ ^]^ Here, we discovered that HSF5, a newly characterized reproduction‐related TF, is crucial for male fertility and that loss of HSF5 leads to meiotic arrest at late pachynema in both humans and mice, revealing the important role of this TF in regulating meiosis.

In summary, the current findings revealed HSF5 as a novel causative gene in meiotic arrest patients that is associated with regulating key events in meiosis, including synapsis, recombination, crossover formation and MSCI. In addition, HSF5 transcriptionally regulates the expression of meiosis‐associated essential genes to play an essential role in the meiosis process. More importantly, we identified that AAV9‐hHSF5‐mediated gene therapy in *Hsf5* KO male mice markedly rescued spermatogenesis, providing a possible direction for the clinical treatment of meiotic arrest patients. Overall, this study provided intriguing insight into the process of meiosis in male reproduction, particularly in humans.

## 4 Experimental Section

### Study Participants

Two infertile siblings from a consanguineous family and their family members were recruited from West China Second University Hospital, Sichuan University, and 1000 fertile (with at least 1 offspring conceived by natural fertilization) Chinese volunteers were enrolled as controls. This study was approved by the Ethical Review Board of West China Second University Hospital, Sichuan University. Informed consent was obtained from each study participant.

### Whole‐Exome Sequencing (WES) and Sanger Sequencing

WES was performed on the patients’ genomic DNA. A whole‐blood DNA purification kit (69 504, Qiagen) was used according to the manufacturer's protocol to extract gDNA from peripheral whole blood samples obtained from participants. An Agilent SureSelect Human All Exon V6 Kit (5190‐6209, Agilent Technologies) was used for exon capture, and then, the DNA sample was sequenced with an Illumina HiSeq X system. The Burrows‒Wheeler Aligner (BWA) was used to compare the resulting sequences with the NCBI human reference genome (UCSC hg19). ANNOVAR was used for functional annotation, and a variety of databases, such as the 1000 Genomes Project, dbSNP, and ExAC, were referenced to filter the data. Variants were excluded according to the following exclusion criteria: (1) minor allele frequency ≥1% in any public database, including gnomAD, ExAC Browser, and 1000 Genomes Project; (2) variants mapping to untranslated regions, noncoding exons, or intronic regions (except typical splice sites); and (3) synonymous variants that were predicted to be tolerated by PolyPhen‐2, SIFT, and Mutation Taster. Furthermore, the filtered candidate pathogenic genes were assessed by the Human Protein Atlas (http://www.proteinatlas.org), which contains tissue‐specific protein expression data, and protein‐coding genes that were primarily or specifically expressed in the testis were included in the research. The candidate pathogenic variant in the patients were validated, their parents and 1000 fertile controls by Sanger sequencing. The primers used for PCR were F, 5′‐TGGCTGATAGGTGAAGAGTAGC‐3′, and R, 5′‐CTGGGCTGCAGTGTATAGGT‐3′.

### Generation of Hsf5‐KO Mice

The animal experiments were approved by the Experimental Animal Management and Ethics Committee of West China Second University Hospital, Sichuan University. All animal procedures complied with the guidelines of the Animal Care and Use Committee of Sichuan University. *Hsf5* mutant mice were generated using CRISPR/Cas9 technology. Briefly, Cas9 mRNA and sgRNA targeting exon 2 of *Hsf5* were injected into mouse embryos at the zygote stage. Cas9 mRNA, sgRNA and mouse zygotes were prepared as previously described.^[^ ^45^ ^]^ DNA from founder mice was PCR amplified and sequenced. Mutant founders were mated with C57/BL6 mice to establish a stable *Hsf5* mutant mouse line. The targeting site for *Hsf5* was ACGTGTACAGATGTGGCGCTGGG. PCR assays and Sanger sequencing was used to identify the frameshift mutation in founder mice. The primers used for PCR were F, 5′‐ACCAGTGGCTGTAGGACAATT‐3′ and R, 5′‐ CCTGTTGGATAGTAGGCTTGGG‐3′.

### Histological Analysis and Immunofluorescence Staining

Testes, epididymis and ovaries were detached and immediately fixed overnight in 4% paraformaldehyde (PFA). For histological analysis, sections were processed and stained with H&E. For immunostaining of the sections, the paraffin sections were dewaxed, rehydrated, and subjected to antigen retrieval by heating in citrate buffer. The sections were then permeabilized in 0.3% Triton X‐100, blocked in 5% bovine serum albumin (BSA) (for PNA staining, 30% normal donkey serum was used to replace 5% BSA), and incubated with primary antibodies at 4 °C overnight. The membranes were then washed with 1× PBST three times, incubated with corresponding secondary antibodies conjugated to Alexa Fluor 488 (1:1000, A21206, Thermo Fisher Scientific) or Alexa Fluor 594 (1:1000, A11005, Thermo Fisher Scientific) at 37 °C for 2 h, and then washed with 1× PBST three times. Then, the cells were stained with 4,6‐diamidino‐2‐phenylindole (DAPI, 28718‐90‐3, Sigma‒Aldrich) for 10 min. The antibodies used in this study targeted HSF5 (1:50, HPA063613, Atlas Antibodies), γH2AX (1:100, ab11174, Abcam), Ki67 (1:100, 27309‐1‐AP, Proteintech), PCNA (1:200, 60097‐1‐Ig, Proteintech), SYCP1 (1:50, ab175191, Abcam), SYCP3 (1:50, ab15093, Abcam), and PNA (1:50, RL‐1072‐5, Vector). Apoptosis was assayed using a TUNEL assay according to the manufacturer's specifications (11 684 795 910, Roche).

### RNA Isolation and Quantitative PCR (qPCR)

The expression profile of *Hsf5* in different mouse tissues and the expression of meiosis‐associated genes were assessed by qPCR. Total RNA was isolated using TRIzol reagent (15 596 026, Thermo Fisher Scientific) and converted to cDNA with SuperScript IV Reverse Transcriptase (18 090 010, Thermo Fisher Scientific) according to the manufacturer's instructions. qPCR was performed using KiCq‐ Start SYBR Green qPCR ReadyMix (KCQS00, Sigma‒Aldrich) on an iCycler qPCR Detection System (Bio‐Rad Laboratories). qPCR data were normalized using the 2−ΔΔCt method. The primer sequences used were shown in Table S2, Supporting Information.

### Cell Culture and Plasmid Construction

HEK293T cells were obtained from the American Type Culture Collection (ATCC CRL‐11268). HEK293T cells were grown in DMEM (11 965 092, Gibco) supplemented with 10% fetal bovine serum (FBS) (F8318, Sigma‒Aldrich). The expression plasmids encoding WT *HSF5* (NM_0 010 80439.2) (pENTER‐Flag‐WT‐*HSF5*) and Mut‐*HSF5* ^p.R196C^ (pENTER‐Flag‐*HSF5* ^p.R196C^) were constructed by Vigene Biosciences (Jinan, China). The plasmids were transfected into HEK293T cells with Lipofectamine 3000 (L3000015, Invitrogen) according to the manufacturer's protocol.

### Western Blotting

Cells and mouse testes were lysed in RIPA buffer (P0013B, Beyotime) supplemented with protease inhibitor cocktail (78 425, Thermo Fisher Scientific). The protein levels of the lysates were quantified by BCA assay (23 227, Thermo Fisher). For SDS‒PAGE separation, lysates were mixed with SDS sample loading buffer (P0015, Beyotime) and boiled for 10 min. Following separation, resolved proteins were transferred to PVDF membranes (IPVH00010, Millipore). The membranes were blocked for 30 min at room temperature and incubated with primary antibodies against HSF5 (1:500) and GAPDH (1:10 000, ab8245, Abcam) at 4 °C overnight. On the next day, the membranes were incubated with secondary antibody diluted in 5% milk and 1× TBST for 1 h at room temperature. Finally, chemiluminescence development and image acquisition were performed in a darkroom.

### Meiotic Studies

For the preparation of nuclear spreads, a drying‐down technique was used.^[^ ^46^ ^]^ Briefly, the testis tubules were disaggregated and chopped with a razorblade in DMEM. Aliquots of the cell resuspension were transferred to slides containing hypotonic buffer (0.5% NaCl, pH 8) and incubated for 15 min to allow the cell to attach to the slide surface. The cells were fixed in 2% PFA with 0.03% SDS for 3 min and 2% PFA for an additional 3 min, rinsed and then allowed to air dry for 10 min. Slides were incubated in 1 × block (10 × stock: 10% goat serum, 3% BSA, 0.05% Triton‐100, in PBS) and then stained overnight at 4 °C with primary antibodies. The slides were rinsed and incubated for 2 h at room temperature with Alexa Fluor 488 (1:1000) or Alexa Fluor 594 (1:1000) diluted in 10× block. The slides were rinsed, soaked briefly in 100 ng/ml DAPI, rinsed again, and allowed to dry in the dark. Coverslips were mounted with antifade media (1 mg ml^−1^ p‐phenylenediamine, 1 × PBS, 80% glycerol).

The following primary antibodies were used: SYCP3 (1:20, sc‐74569, Santa Cruz), SYCP1 (1:50, NB300‐229, Novus), RAD51 (1:200, PC130, Millipore), MLH1 (1:100, 4C9C7, CST), MSH4 (1:100, ab58666, Abcam), γH2AX (1:1000), TEX12 (1:50, 17068‐1‐AP, Proteintech), p‐RNAP (1:50, ab193467, Abcam), HORMAD1 (1:50, 13917‐1‐AP, Proteintech), RPA2 (1:50, ab76420, Abcam), and H3k4me3 (1:50, ab213224, Abcam). A mouse monoclonal antibody against H1t (1:50) and a rabbit monoclonal antibody against TEX11 (1:50) were obtained from the laboratory of Prof. Qinghua Shi, University of Science and Technology of China, and a rabbit monoclonal antibody against MLH3 (1:50) was obtained from the laboratory of Prof. Mengcheng Luo, Wuhan University.

### Chromatin Immunoprecipitation Sequencing (ChIP‐seq)

Chromatin samples were prepared with a Chromatin IP Kit (9005S, CST) according to the manufacturer's guidelines. Human testicular tissue was digested, and the enriched cells were cross‐linked with 1% formaldehyde for 10 min at room temperature. This process was stopped by the addition of glycine solution. Chromatin was fragmented by sonication using a Covaris‐S220 sonicator in ChIP SDS lysis buffer (50 mM Tris–Cl, pH 8; 10 mM EDTA, pH 8; 1% SDS). The cross‐linked chromatin was incubated with HSF5 antibodies in ChIP dilution buffer (50 mM HEPES, pH 7.5, 155 mM NaCl, 1.1% Triton X‐100, 0.11% Na‐deoxycholate) supplemented with protease inhibitors overnight. Crosslinking was reversed overnight at 65 °C, and DNA was extracted with phenol/chloroform/isoamyl alcohol.

For ChIP‐seq, DNA was amplified according to the Illumina ChIP Sequencing Sample Preparation Guide using adaptors and primers. Deep sequencing was performed at the Computational Biology Omics Core of the CAS‐MPG Partner Institute using Illumina HiSeq X‐10 (2 × 150). ChIP‐seq reads were aligned to the human genome using BOWTIE software (Bowtie2 version 2.1.0). The aligned reads were filtered to eliminate those that exhibited low mapping quality and duplications arising as an artifact of sample amplification or sequencing. Peak calling was performed with MACS2 (version 1.4.2) on the ChIP file against the input file. Genome‐wide normalized signal coverage tracks were created by bamCoverage in deepTools (version 3.3.0) and visualized in the Integrative Genomics Viewer (IGV version 2.5.0). Peaks were annotated to the genomic region and the nearest genes (TSS within 2 kb) using the Bioconductor package ChIPSeeker (version 1.16.1). Peaks overlapping by at least 1 nt with unique gene model promoters (± 2 kb of each unique gene model TSS) were considered to be localized to those promoters. Motif analysis was performed to measure the enrichment of DNA binding sequences within 400 bp regions centred on the peaks using Centrimo (known) and MEME (*de novo*). Precipitated DNA was amplified for deep sequencing or analyzed by qPCR. PCR and qPCR were performed to amplify the target regions of the *TEX15*, *SYCP1*, *SYCP2*, *SYCE3*, *MDC1*, *HFM1*, *MLH3*, *MSH5*, *HSF2* and *HSF5* promoters to which HSF5 binds. The qPCR data were analyzed by the fold enrichment method and percent input method simultaneously. In the percent input method, the value of target DNA fragments that were enriched in testis tissue was normalized to the value of 1% of the input DNA. Each assay was performed in triplicate to confirm the reproducibility of the results. The primers used were listed in Table S3, Supporting Information.

### Dual‐Luciferase Reporter Assay

The *TEX15*, *SYCP1*, *SYCP2*, *SYCE3*, *MDC1*, *HFM1*, *MLH3*, *MSH5*, *HSF2* and *HSF5* promoter fragments were amplified and cloned into the pGL3‐basic luciferase reporter vector (Promega). Seven plasmids containing the above promoter regions were separately cotransfected with the Flag‐WT‐*HSF5* plasmid or individually transfected into HEK293T cells; the pGL3‐control plasmid served as a positive control, and the pGL3‐basic plasmid served as a negative control. After 48 h, the cells were harvested for the dual‐luciferase assay (E1910, Promega). The firefly luciferase values were normalized to the Renilla luciferase activity before statistical analyses. The primers used for promoter region amplification were listed in Table S4, Supporting Information.

### Electrophoretic Mobility Shift Assay (EMSA)

First, HEK293T cells were transfected with WT‐*HSF5* plasmids. Nuclear extracts were obtained using a nuclear protein extraction kit (P0027, Beyotime) according to the manufacturer's instructions. Double‐stranded oligonucleotides were obtained by annealing equal amounts (0.1 mg) of the complementary single‐stranded oligonucleotides by heating to 95 °C for 5 min and then gradually cooling to room temperature. Then, 0.01 µmol of digoxigenin‐labeled oligonucleotide probes was incubated with nuclear extracts in DNA binding buffer [10 mM Tris‐HCl (pH 7.5), 1 mM MgCl_2_, 50 mM NaCl, 0.5 mM EDTA, 4% glycerol, and 0.5 mM 2,3‐dihydroxy‐l,4‐dithiobutane (DTT)] and 1 µg of poly(dI‐dC). A competition assay was performed using a 200‐fold excess of cold probes or cold mutated probes (2 µmol), which were preincubated with the reaction mixture before the addition of biotin‐labeled probes. To determine the specificity of the nuclear protein‐bound sites, a supershift assay was performed with 2 mg of HSF5 antibody. After incubation for 30 min, the DNA‒protein complexes were separated by 6.0% nondenaturing PAGE and transferred to a nylon membrane. DNA was crosslinked by UV irradiation for 10 min. The nitrocellulose membrane was evaluated by the addition of a streptavidin–horseradish peroxidase conjugate and a chemiluminescent substrate. Then, the nitrocellulose membrane was imaged with an e‐BLOT luminescent image analyzer. The label probe was incubated with the nuclear protein, and the protein bound to the label probe to form a protein–probe complex. This complex had a large molecular weight and migrated slowly on gel electrophoresis, while the label probe without binding protein migrated more rapidly. The protein–probe complex formed a band at the front of the membrane, indicating that there was interaction between the protein and the target probe. In competition experiments, a cold probe (WT probe without biotin modification) was used to determine the specificity of the protein–probe binding. The cold probe contains the same DNA sequence as the label probe, which can interfere with the binding between the probe and the protein.

### RNA Sequencing (RNA‐seq)

Total RNA was extracted from the samples with TRIzol reagent. Strand‐specific libraries were prepared using the TruSeq Stranded Total RNA Sample Preparation Kit (RS‐122‐2001, Illumina) according to the manufacturer's instructions and then submitted to the Illumina HiSeq X Ten system. RNA‐seq library preparation and sequencing were performed at Novogene (Beijing, China). The number of aligned reads for individual genes were counted using HTSeq followed by DESeq2 normalization to evaluate gene expression as normalized counts per million. Significantly differentially expressed genes were identified as those with a *P* value or false discovery rate (FDR) value above the threshold and a fold change >1.5 using DESeq2 software.

### Isolation of Mouse Spermatogenic Cells

Pachytene spermatocytes were isolated and enriched through cell diameter/density at unit gravity using the STA‐PUT velocity sedimentation method, as previously described.^[^ ^47^ ^]^ In brief, mouse testes were extracted and placed into 1640 medium and digested with 0.5 mg ml^−1^ collagenase (17 018 029, Gibco) for 15 min and then with 0.5 mg/ml trypsin (15 090 046, Gibco) containing 1 µg ml^−1^ DNase I (18 047 019, Gibco) for 30 min. The released cells were resuspended in 25 ml of 0.5% BSA solution and passed through an 80 mm mesh to remove cell aggregates. Then, the samples were sedimented at unit gravity through a 2–4% BSA gradient generated in a medium‐sized STA‐PUT chamber (ProScience, Inc.) for gradient separation.

### Adeno‐Associated Virus Vector Packaging

AAV9‐CAG‐mScarlet and AAV9‐hHSF5‐P2A‐mScarlet were obtained from pAAV‐CAG‐eGFP (188 039, Addgene). For the construction of AAV9‐CAG‐mScarlet, mScarlet was synthesized by Youkang Company according to the reference sequence from pLBS‐mScarlet (29 337, Addgene) and inserted into pAAV9‐CAG‐eGFP vector cut via *KpnI/AscI* by using the ClonExpress II One Step Cloning Kit (C112‐01, Vazyme). hHSF5‐P2A was synthesized by Youkang Company according to the reference sequence from NCBI (NM_0 010 80439) and inserted into AAV9‐CAG‐mScarlet via *KpnI* by using the ClonExpress II One Step Cloning Kit (C112‐01, Vazyme).

HEK293T cells in ten 15 cm plates were transfected with 70 µg of the AAV2 genome (carrying the transgene), 70 µg of pAAV2/9n (112 865, Addgene) and 200 µg of pAdDeltaF6 (112 867, Addgene) in 18 mL of Opti‐MEM (31 985 070, Gibco) and 1.7 mL of PEI (49553‐93‐7, Sigma‒Aldrich) for 72 h. After transfection, the cells were harvested and suspended in AAV lysis buffer (1 mM MgCl_2,_ 100 mM NaCl, 20 mM Tris, pH 8.0) and lysed with Benzonase (SC‐391121, Santa Cruz) in four −80 °C to 37 °C cycles. AAV was purified through four gradients of OptiPrep (92339‐11‐2, Sigma‒Aldrich) with an ultracentrifuge (60,000 rpm with rotor T70i, 2 h at 4 °C) and concentrated with PBS + 0.001% F68 (24 040 032, Thermo Fisher) three times. The AAV titer was quantitated via qPCR using the AAV9‐CAG‐mScarlet plasmid with the ITR primer to generate a standard curve.

### Gene Delivery in Animal Models

Three‐week‐old male mice were anesthetized, and their testes were removed from the abdominal cavity. Approximately 7 µl of AAV9‐hHSF5‐P2A‐mScarlet solution mixed with indicator dye (0.3% trypan blue) was injected into the rete testis using a glass capillary needle under a stereomicroscope (OPMI VARIO S88, Carl Zeiss AG). An equal amount of AAV9‐control was injected into the other testis. The testes were replaced, and the incision was sutured. Assessment of spermatogenesis was performed 5 weeks later.

### Statistical Analysis

Statistical analyses were performed using GraphPad Prism 8.4.0 software and SPSS 17.0 software. All the data were presented as the mean ± SEM. A *P* value less than 0.05 was considered to indicate statistical significance. The statistical significance of differences between two groups was calculated using an unpaired, parametric, two‐sided Student's *t* test.

### Ethical Approval

The animal experiments (033) and human subjects (040) were approved by the Experimental Animal Management and Ethics Committee of West China Second University Hospital, Sichuan University. All animal procedures complied with the Animal Care and Use Committee of Sichuan University.

## Conflict of Interest

The authors declare no conflict of interest.

## Author Contributions

M.L., L.W., Y.L., E.Z., G.S., X.J. contributed equally to this work. Y.S. designed and supervised the study experiments. Y.Y., X.J., D.L. and G.Z. collected data and conducted the clinical evaluations. M.L. and G.S. performed experiments and analyzed most of the data. L.W., X.Z., B.W. and N.O. generated and bred the CRISPR mice. Y.L. and Y.Z. constructed AAVs. E.Z. performed testicular microinjection of AAVs. C.J., T.R., X.W., X.Z., S.D. and R.Z. performed the experiments. Y.S. and M.L. wrote the manuscript, with input from others. H.L. and L.Y. provided valuable advice and financial support for this research. All authors revised and approved the article.

## Supporting information

Supporting Information

## Data Availability

The data that support the findings of this study are available from the corresponding author upon reasonable request.
