# Supplementary material for: HSF5 Deficiency Causes Male Infertility Involving Spermatogenic Arrest at Meiotic Prophase I in Humans and Mice
Source: Adv Sci (Weinh). 2024 Jul 3;11(33):2402412. doi: 10.1002/advs.202402412 (PMC11434121; doi:10.1002/advs.202402412)
Supplement: Supplementary file 1 — Supporting Information [file ADVS-11-2402412-s001.pdf]

ADVANCED SCIENCE
Open Access

Supporting Information

for *Adv. Sci.*, DOI 10.1002/advs.202402412

HSF5 Deficiency Causes Male Infertility Involving Spermatogenic Arrest at Meiotic Prophase I in Humans and Mice

Mohan Liu, Lingbo Wang, Yifei Li, Erlei Zhi, Gan Shen, Xiaohui Jiang, Dingming Li, Xinya Zhao, Tiechao Ruan, Chuan Jiang, Xiang Wang, Xueguang Zhang, Yanjiang Zheng, Bangguo Wu, Ningjing Ou, Guicheng Zhao, Siyu Dai, Ruixi Zhou, Li Yang, Yihong Yang*, Hanmin Liu* and Ying Shen**

Supplementary information

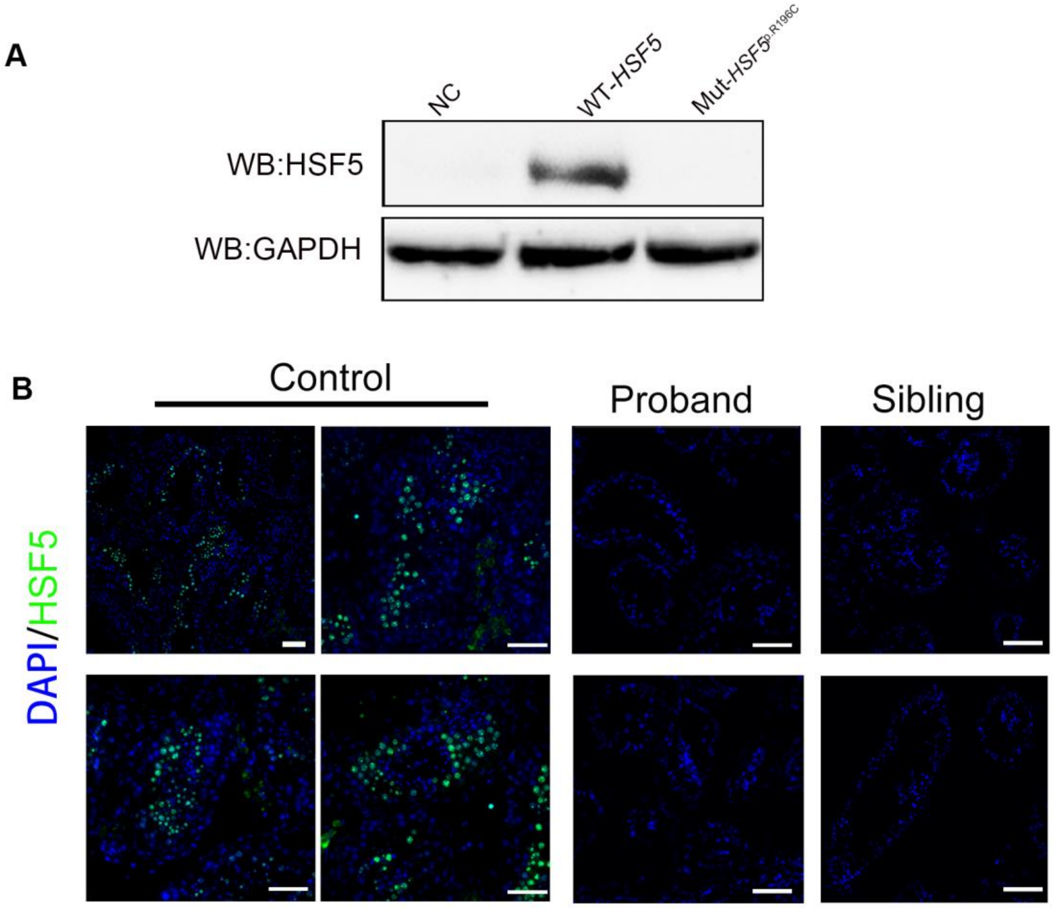

Figure S1. Expression analysis of the *HSF5*^{R196C} variant. A) Western blotting analysis detected no HSF5 expression in the cells transfected with the mut-*HSF5*^{R196C} plasmid. Three independent experiments were performed. B) Absent expression of HSF5 was observed in the testes from patients by immunofluorescence staining. Three independent experiments were performed (Blue, DAPI; green, HSF5; scale bars, 100 μm).

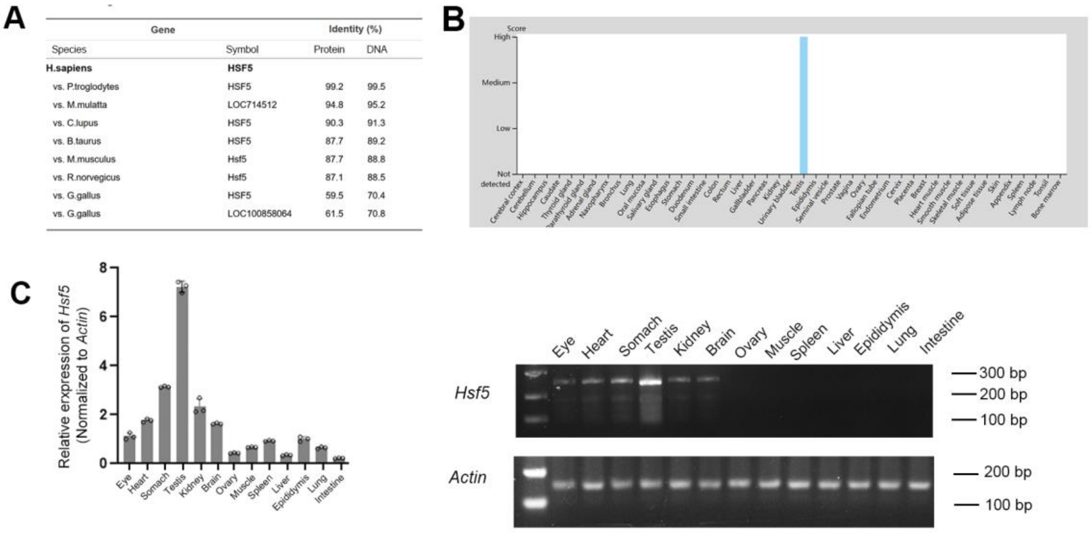

Gene		Identity (%)	
Species	Symbol	Protein	DNA
**H.sapiens**	**HSF5**		
vs. P.troglodytes	HSF5	99.2	99.5
vs. M.mulatta	LOC714512	94.8	95.2
vs. C.lupus	HSF5	90.3	91.3
vs. B.taurus	HSF5	87.7	89.2
vs. M.musculus	Hsf5	87.7	88.8
vs. R.norvegicus	Hsf5	87.1	88.5
vs. G.gallus	HSF5	59.5	70.4
vs. G.gallus	LOC100858064	61.5	70.8

Figure S2. The tissue-specific expression pattern of HSF5/*Hsf5*. A) Homology of *HSF5*/HSF5 among different species (https://www.ncbi.nlm.nih.gov/homologene?cmd=Retrieve&dopt=AlignmentScores&list_uids=52701). B) The relative expression level of HSF5 in different human tissues (https://www.proteinatlas.org/ENSG00000176160-HSF5/tissue). C) qPCR revealed that *Hsf5* is predominantly expressed in the mouse testes (N = 3 biologically independent WT mice; error bars, mean ± SEM).

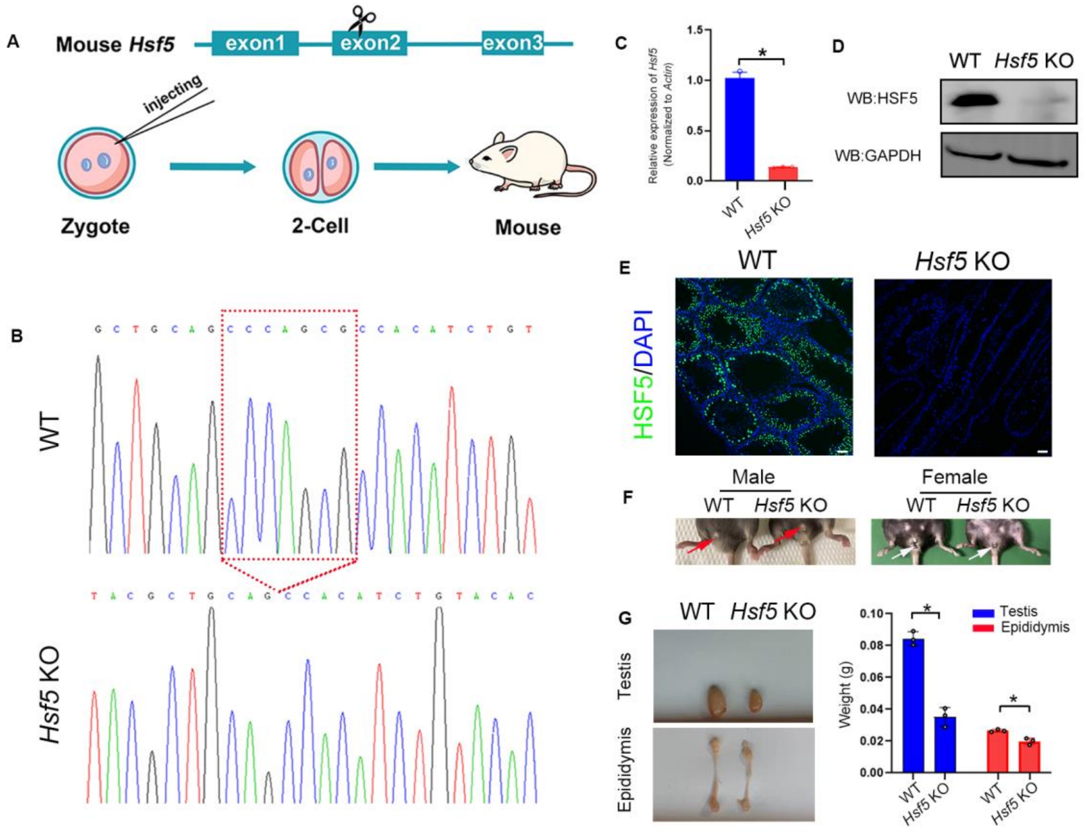

Figure S3. Targeted inactivation of *Hsf5* in mice. A) Schematic illustration of the targeting strategy for generating *Hsf5* KO mice. B) Results of PCR-seq genotyping using tail DNA from *Hsf5* KO mice. The red line designates the deleted bases in the KO mice. C) qPCR analysis of *Hsf5* expression in WT and *Hsf5* KO mouse testes (N = 3 biologically independent WT mice and KO mice; two-sided Student's *t* test; * $P<0.05$; error bars, mean ± SEM). D) Western blotting showed the absence of HSF5 in *Hsf5* KO testes (N = 3 biologically independent WT mice and KO mice). E) Immunofluorescence staining of HSF5 in testis sections from WT and *Hsf5* KO mice (N = 3 biologically independent WT mice and KO mice; scale bars, 50 μm). F) Representative photographs of the reproductive organs of WT mice and KO mice. The red arrows indicate the penises, and the white arrows indicate the female reproductive organs (N= 3 biologically independent WT mice and KO mice). G) Dramatic size reduction in testes

and epididymides from the adult *Hsf5* KO mice (N = 3 biologically independent WT mice and KO mice; two-sided Student's *t* test; * $P<0.05$; error bars, mean ± SEM).

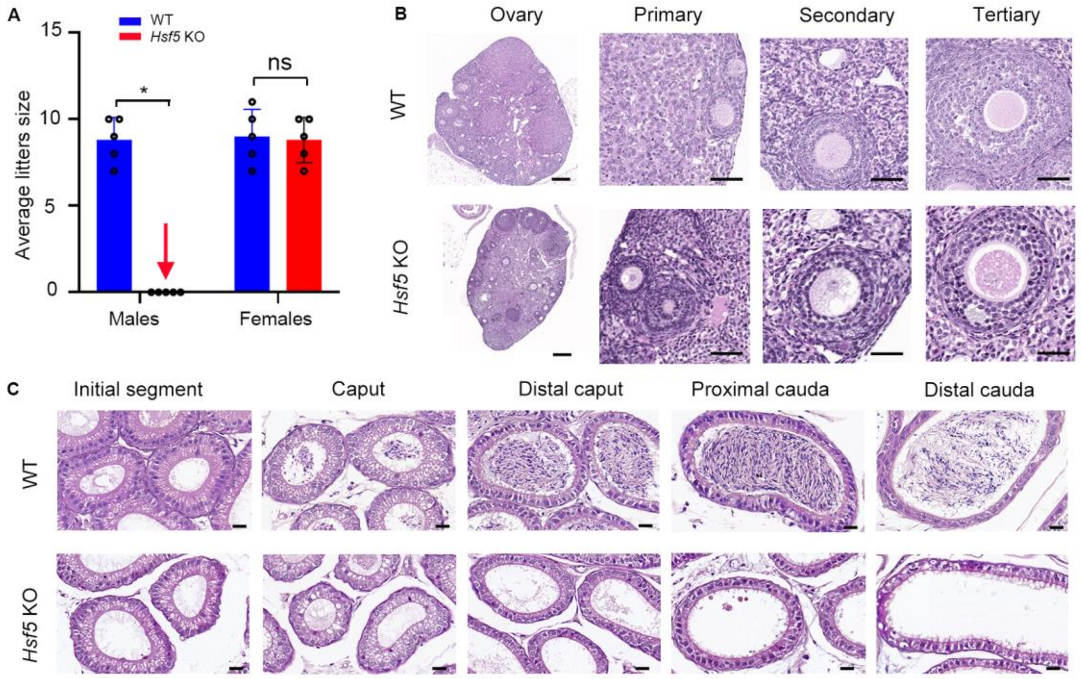

Figure S4. Fertility of *Hsf5* KO mice. A) Fertility in WT and *Hsf5* KO mice. *Hsf5* KO males were completely sterile, while the females showed normal fertility (N = 5 biologically independent WT mice and KO mice; two-sided Student's *t* test; ns, not significant; * $P<0.05$; error bars, mean ± SEM). B) H&E staining of sections from WT and *Hsf5* KO mouse ovaries (8 weeks old) (N = 3 biologically independent WT mice and KO mice; scale bars, 50 μm). C) No spermatozoa were observed in individual fields of the epididymides in *Hsf5* KO mice compared to WT mice (8 weeks old) (N = 3 biologically independent WT mice and KO mice; scale bars, 100 μm).

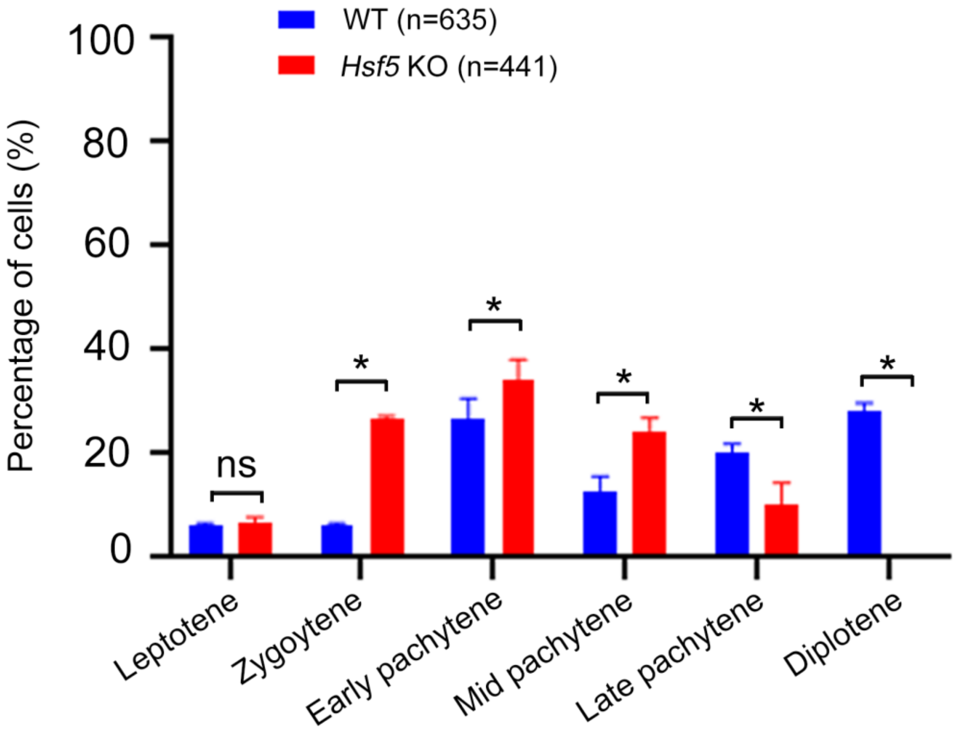

Figure S5. Percentages of substages of spermatocytes in *Hsf5* KO mice. Quantification of the proportion of spermatocytes from Fig. 4 (N = 3 biologically independent WT mice and KO mice; n, the total number of nuclei analyzed; two-sided Student's t test; * P<0.05; error bars, mean ± SEM).

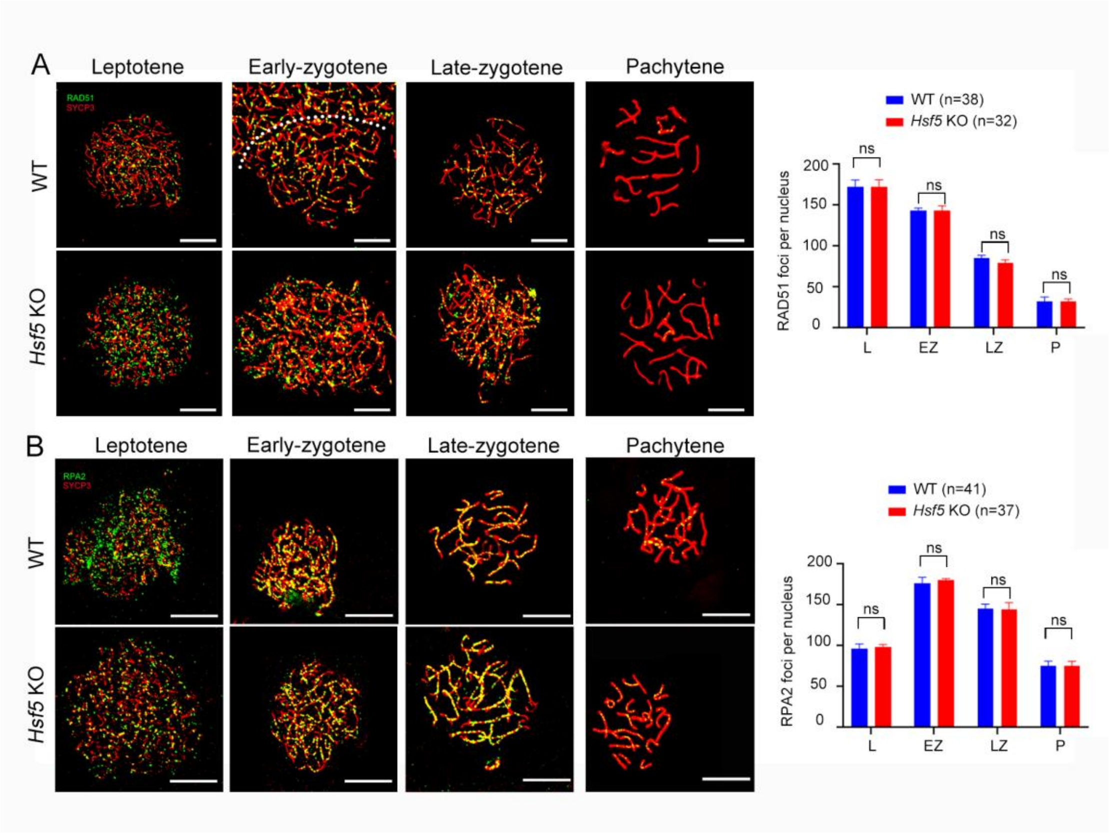

Figure S6. HSF5 is dispensable for early recombination. A) Immunofluorescence staining showed RAD51 foci in spread spermatocytes from 8-week-old WT and *Hsf5* KO mice. RAD51 focus counts were not significantly different between spermatocytes from WT and *Hsf5* KO mice (N = 3 biologically independent WT mice and KO mice; n, the total number of nuclei analyzed; two-sided Student's t test; ns, not significant; error bars, mean ± SEM; green, RAD51; red, SYCP3; scale bars, 10 μm). B) RPA2 signals were analyzed in WT and *Hsf5* KO spermatocytes. The RPA2 foci exhibited no significant difference between spermatocytes from WT and *Hsf5* KO mice (N = 3 biologically independent WT mice and KO mice; n, the total number of nuclei analyzed; two-sided Student's t test; ns, not significant; error bars, mean ± SEM; green, RPA2; red, SYCP3; scale bars, 10 μm).

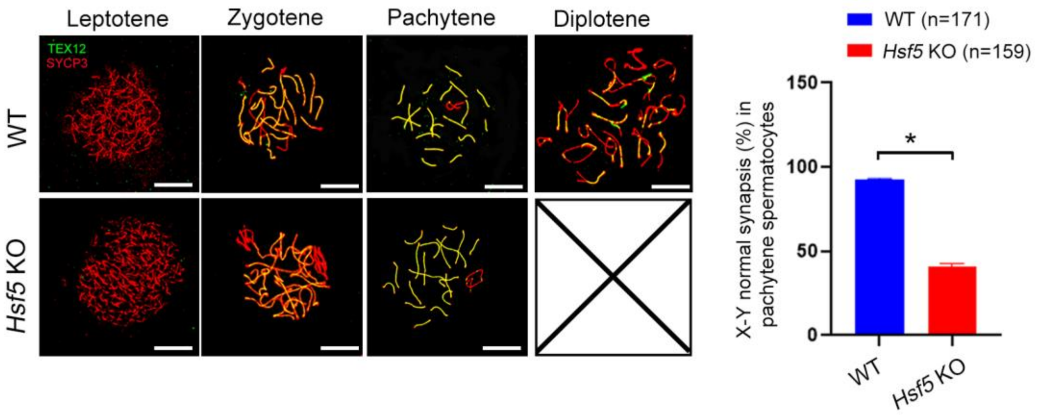

Figure S7. *Hsf5* KO spermatocytes fail to complete sex chromosome synapsis. Immunofluorescence staining of TEX12 and SYCP3 was analyzed in spread spermatocytes from 8-week-old WT and *Hsf5* KO mice. Quantification of normal synapsis in spermatocytes from WT and *Hsf5* KO mice (N = 3 biologically independent WT mice and KO mice; n, the total number of nuclei analyzed; two-sided Student's t test; * $P<0.05$; error bars, mean ± SEM; green, TEX12; red, SYCP3; scale bars, 10 μm).

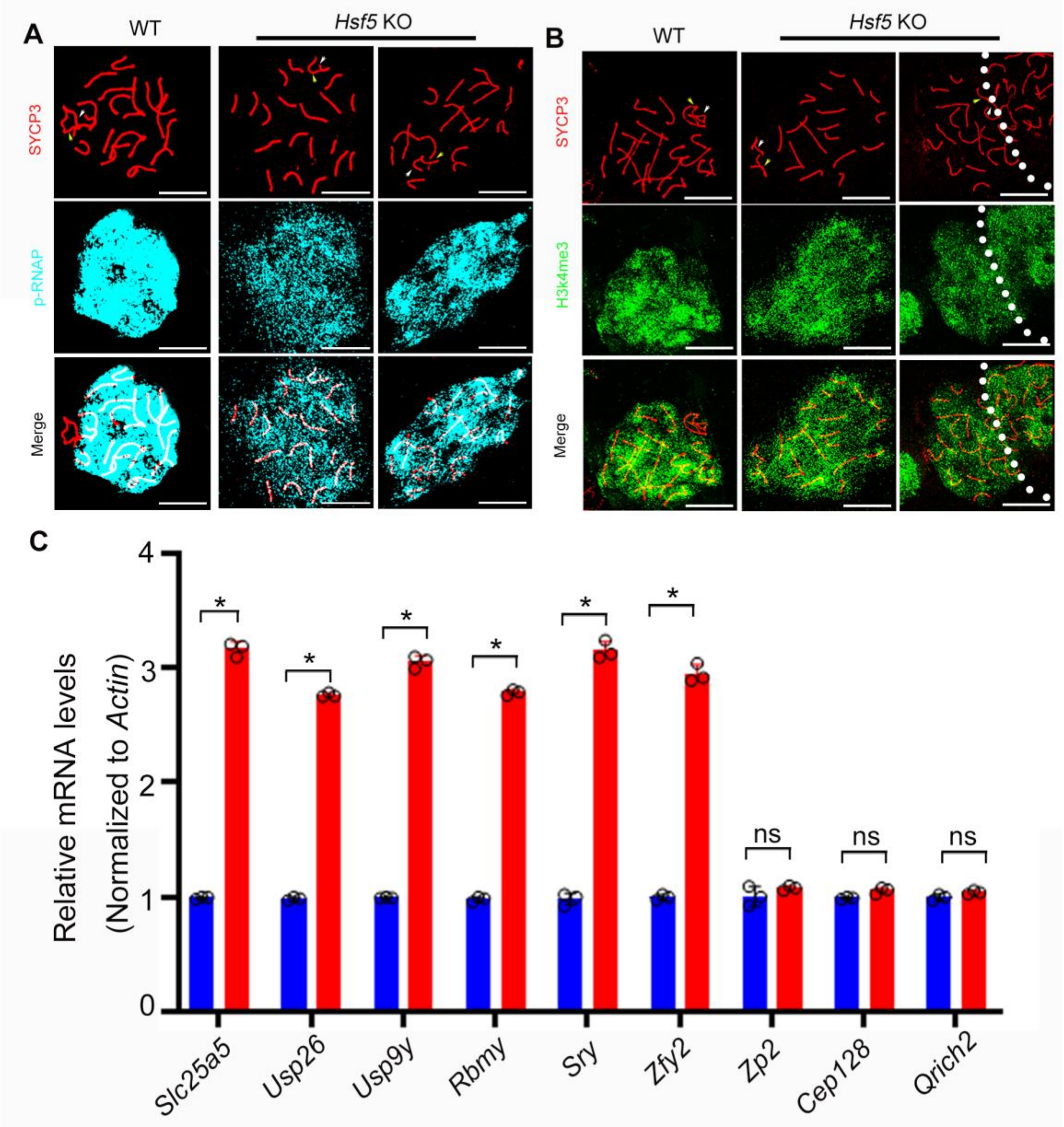

Figure S8. *Hsf5* KO mice show impaired MSCI. A) Spread spermatocytes from 8-week-old WT and *Hsf5* KO mice stained for SYCP3 and p-RNAP. In WT spermatocytes, p-RNAP staining was absent from the sex chromosomes, whereas it was present in that of *Hsf5* KO spermatocytes. Yellow arrow indicates X chromosome and white arrow indicates Y chromosome (N = 3 biologically independent WT mice and KO mice; sky blue, p-RNAP; red, SYCP3; scale bars, 10 μm). B) Spread spermatocytes from 8-week-old WT and *Hsf5* KO mice stained for SYCP3 and H3k4me3. H3k4me3 was not excluded in the sex chromosomes of *Hsf5* KO pachytene spermatocytes. Yellow arrow

indicates X chromosome and white arrow indicates Y chromosome (N = 3 biologically independent WT mice and KO mice; green, H3k4me3; red, SYCP3; scale bars, 10 μm). C) qPCR analysis of gene expression in sex chromosomes and autosomes in total RNA from isolated pachytene spermatocytes from WT and *Hsf5* KO testes (N = 3 biologically independent WT mice and KO mice; two-sided Student's *t* test; * $P<0.05$; ns, not significant; error bars, mean ± SEM).

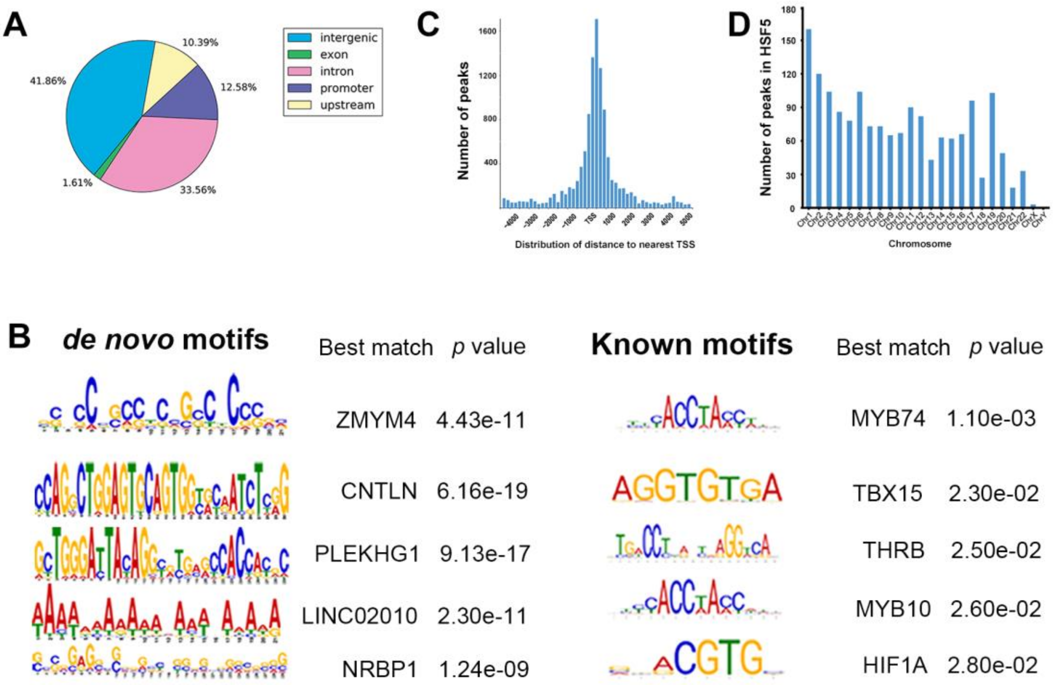

Figure S9. The DNA-binding activity of HSF5 in testes. A) Genomic localization of HSF5 occupancy sites by ChIP-seq. B) Identification of HSF5 binding motif(s) in known and *de novo* motif analysis. C) Density distribution of HSF5 ChIP-seq peaks. D) The HSF5 binding strength for each chromosome.

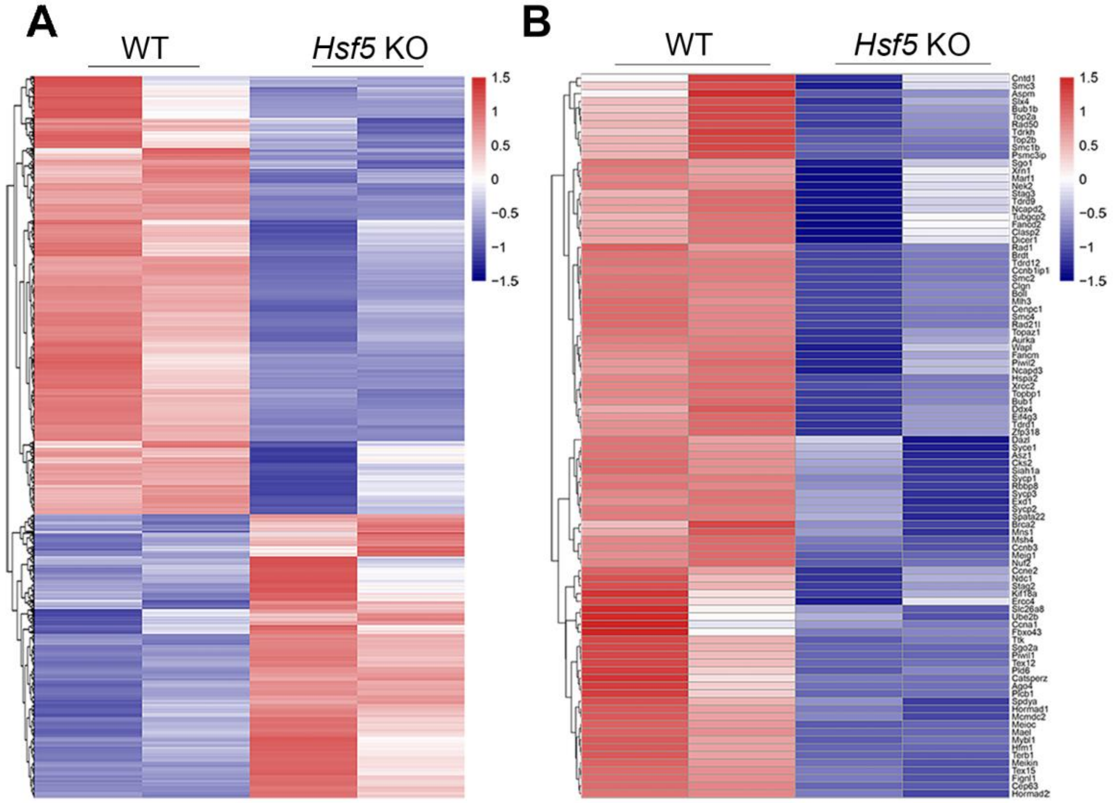

Figure S10. The transcriptome of *Hsf5* KO testes is substantially different from that of WT testes. A) Heatmap of genes differentially expressed in testes between WT and *Hsf5* KO mice at 20 dpp as determined by RNA-seq (N = 2 biologically independent WT mice and KO mice). B) Heatmap of genes associated with spermatogenesis differentially expressed between WT and *Hsf5* KO male mice by RNA-seq analysis (N = 2 biologically independent WT mice and KO mice).

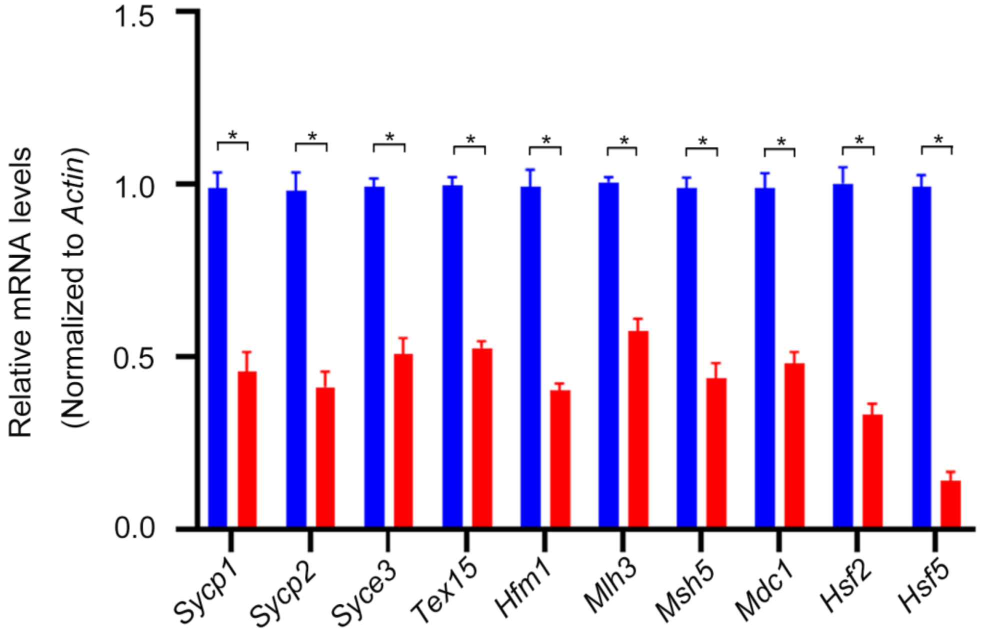

Figure S11. Downregulated mRNA expression of meiosis-related genes is detected in *Hsf5* KO testes. The mRNA levels of *Sycp1*, *Sycp2*, *Syce3*, *Tex15*, *Mdc1*, *Hfm1*, *Mlh3*, *Msh5*, *Hsf2* and *Hsf5* in the testes of WT and *Hsf5* KO mice (N = 3 biologically independent WT mice and KO mice; two-sided Student's *t* test; * *P*<0.05; error bars, mean ± SEM).

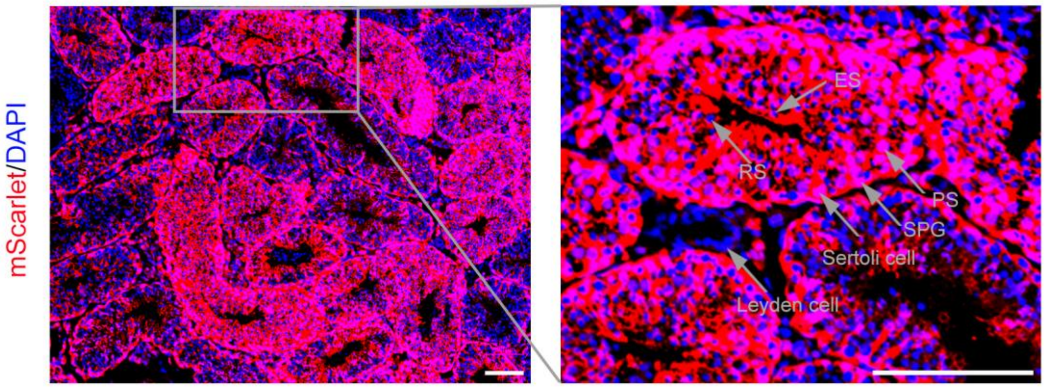

Figure S12. Optimal AAV concentration. WT mouse testes after microinjection with

$8*10^{\wedge 10}$ gc mScarlet-expressing AAV9 for one week. SPG, spermatogonium; PS, primary spermatocyte; RS, round spermatid; ES, elongating/elongated spermatid (N = 3 biologically independent 8-week-old WT mice; blue, DAPI; red, mScarlet; scale bars, 125 μm).

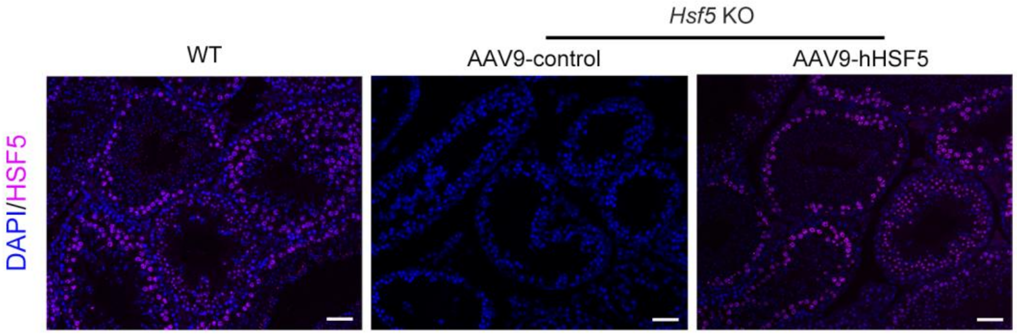

Figure S13. Testicular injection of AAV-hHSF5 rescues HSF5 expression in *Hsf5* KO mice. Immunofluorescence staining of HSF5 in the testes of *Hsf5* KO mice microinjected with AAV9-control and AAV9-hHSF5 compared to WT mice with AAV9-control (N = 3 biologically independent WT mice and KO mice; scale bars, 125 μm).

Table S1. Analysis of c.586C>T variant in *HSF5*.

**Variant**	cDNA mutation	c.586C>T
	Protein changes	p.R196C
	Mutation type	Missense
	Genotype	Homozygous
**Allele frequency**	in ExAC Browser	0.00000845
	GnomAD	0.000004053
	1000 Genomes Project	NA
**Function prediction**	SIFT	Deleterious
	Polyphen-2	Probably damaging
	CADD	Deleterious

	Mutation Taster	Disease_causing

Note: RefSeq accession number of *HSF5*: NM_001080439.2; NA: Not available

Table S2. Overview of the primer sequences used in qPCR in mice.

Primers	Sequences
β-actin	F: 5'-CCCAGGCATTGCTGACAGG-3'
(141 bp)	R: 5'-TGGAAGGTGGACAGTGAGGC-3'
Hsf5	F: 5'-TTTTGCACGAAGTCAGTTGGG-3'
(288 bp)	R: 5'-GGTTTCCGAAGTCGTTCTGT-3'
Sycp1	F: 5'-CTGACAACGGCCCAGGAG-3'
(123 bp)	R: 5'-AGTTGGAGTCTCCTCCCGC-3'
Mdc1	F: 5'-CTGTCCCTGAACTGGCTGTACCAG-3'
(426 bp)	R: 5'-GGTAGATGACATTTCCAAATTGGA-3'
Tex15	F: 5'-TCATACCCACTGGTAACACAG-3'
(220 bp)	R: 5'-GGCAAAACATCACTCAAACCTG-3'
Hsf2	F: 5'-AAGGCCAGGATGACCTGTTG-3'
(441 bp)	R: 5'-TCAGTCCTGCTGTGTGGAAC-3'
Sycp2	F: 5'-AGGATGAGATCACTACACCTAGC-3'
(208 bp)	R: 5'-GGTGACGCAGCATAATCCATT-3'
Syce3	F: 5'-CTGATTCCGATCCTGGGGAAA-3'
(116 bp)	R: 5'-GGTTGCCTGCACTGAGATTTT-3'
Mlh3	F: 5'-CAGGACAGCAACAACAGTGC-3'
(176 bp)	R: 5'-TGATGCATTTGGATGGGGCT-3'
Hfm1	F: 5'-GTTGCACAAAAGCCGGAAGT-3'
(300 bp)	R: 5'-ACCCAGAACTTCTTTGTCACG-3'
Msh5	F: 5'-CCTTCTTTCCATTCCCCGCT-3'
(319 bp)	R: 5'-GGGAGAGTAATGCGGTCTCG-3'
Cep128	F: 5'-GATGCTGAAGGCAGAAAGC-3'

(178 bp)	R: 5'-CTCCTTCTTGTCTAGTTCCTCC-3'
Qrich2	F: 5'-CTCTGTCTCTGAGGCGTCTC-3'
(206 bp)	R: 5'-GCAGACTGACTTGGTGGCTC-3'
Usp26	F: 5'-GCACAAGTGCACAGGAGTTG-3'
(462 bp)	R: 5'-ATTCATGCCACTCTAGGCCG-3'
Slc25a5	F: 5'-AGGGCATCATAGACTGCGTG-3'
(170 bp)	R: 5'-CTGGGTCCTCTTGTCCACAC-3'
Usp9y	F: 5'-AAGCCGTTTGGACAGTGCTA-3'
(392 bp)	R: 5'-TCTGTCATTCGCTCAACGGT-3'
Rbmy	F: 5'-TGAGGCACCATCTGCAAGAG-3'
(208 bp)	R: 5'-ATGCCCTCTTTCCTGTCTGC-3'
Zfy2	F: 5'-TAGGAGCTGATGCAGTACACA -3'
(443 bp)	R: 5'-TGAAATGTCGGCTGTCAAAGG-3'
Sry	F: 5'-GCCTCATCGGAGGGCTAAAG-3'
(91 bp)	R: 5'-GTCCCACTGCAGAAGGTTGT-3'
Zp2	F: 5'-GCATCACTGAGGAGCAAACG-3'
(144 bp)	R: 5'-GAAAAAGGTACCACAGCACCAG-3

Table S3. Overview of the primer sequences used in ChIP-PCR and ChIP-qPCR.

Primers	Sequences
SYCP1	F: 5'-AGAGCCACTTTGCCTACGTC-3'
(318 bp)	R: 5'-ACGGCTAAATAACCGTCGCT-3'
SYCP2	F: 5'-TTTTGCACGAAGTCAGTTGGG-3'
(288 bp)	R: 5'-GGTTTCCGAAGTCGTTCTGT-3'
SYCE3	F: 5'-CTCCTCCCAGCCAGAGCTTTCTG-3'
(515 bp)	R: 5'-CTCATTGGGTGTCCTCGGGAAAAG-3'
MDC1	F： 5'-GAGGTGGGGACCAAAGATGG-3'
(379 bp)	R： 5'-CTTCACACTCACCCACCTCC-3'

TEX15	F：5'-CCCTAAGTGCACTGATTGCTTTTGG-3'
(346 bp)	R：5'-CGTGGCAACACATCAGCTAATTTTC-3'
HSF5	F：5'-TCTGCCGGGGTGTTCTAAGT-3'
(157 bp)	R：5'-CCTTTGGTCCCCAGAACGAC-3'
HSF2	F: 5'- TGAACCCCCTGAATAACCGC-3'
(438 bp)	R：5'-TCGCTGAGGTAAAGATCGCC-3'
MSH5	F: 5'-GTTGGGAAGAGCTGGGCAAG-3'
(291 bp)	R：5'-GAAGCTTGAGGCTCTCGTGG-3'
HFM1	F: 5'-TAAGCCGAGCTCCAAGCAAT-3'
(204 bp)	R：5'-TGGGGGTAGAGAAGCAGGTT-3'
MLH3	F: 5'-GTGTTAGAAGAGACCCCGGC-3'
(217 bp)	R：5'-CTTGGCTGGAACAACTGGTG-3'

Table S4. Overview of the primer sequences used in plasmid construction for dual-luciferase reporter assay.

Primers	Sequences
TEX15 promoter	F: 5'-TTACATGGCATTATTTGGCACTCA -3'
(2063 bp)	R: 5'-GGGAGTCTCAGACGCTACTCT-3'
SYCP2 promoter	F: 5'-AACGACTTCGGAAACCGAGA-3'
(1917 bp)	R: 5'-GGCGTTCATCAGGAGAGCATA-3'
SYCP1 promoter	F: 5'-CACTTAATCCTCACACAGAACGC-3'
(1813 bp)	R: 5'-TGCCTACACAATAATGCTCACTG-3'
SYCE3 promoter	F：5'-CCTTTCCGTTAGCGAACCCT-3'
(1802 bp)	R：5'-GGGAAGACCAGCCTGACATC-3'
HSF2 promoter	F：5'-GTGCAAACCACACTTTGCCT-3'
(1942 bp)	R：5'-CCCCTACTCAACTCCAAGCA-3'
MDC1 promoter	F：5'-CCCCAGTCTTCGAACACACT-3'

(1865 bp)	R： 5'-GGTGGGGACAGCCTTTAAGA-3'
HSF5 promoter	F: 5'-GGGGAAGTTGTTGGGGTTGA-3'
(751 bp)	R： 5'-TGGGCAGGCACACAAGATAG-3'
MSH5 promoter	F: 5'- GAATCGTTGCTTCCGAACCG-3'
(922 bp)	R： 5'-ACATCTGCAATCCCAGAGAGC-3'
HFM1 promoter	F: 5'-GGCCTGGGAAAGGACCATAC-3'
(937 bp)	R： 5'-GGTTGTGGGGGTAGAGAAGC-3'
MLH3 promoter	F: 5'-CTCCATCAGTGAGAGCCACG-3'
(1031 bp)	R： 5'-TCTCAGCCTTTTCCAGCTCC-3'
